# Metastases with definitive pathological diagnosis but no detectable primary tumor: A surveillance epidemiology and end results‐based study

**DOI:** 10.1002/cam4.2496

**Published:** 2019-08-13

**Authors:** Lianyuan Tao, Haibo Yu, Yadong Dong, Guanjing Tian, Zhiyuan Ren, Deyu Li

**Affiliations:** ^1^ Department of Hepatobiliary Surgery Henan Provincial People's Hospital People's Hospital of Zhengzhou University, School of Clinical Medicine Henan University Zhengzhou China

**Keywords:** cancer of unknown primary site, metastasis, prognosis, SEER, survival

## Abstract

**Background:**

This study investigates the characteristics of a special type of cancer of unknown primary site (CUP, type 2), which is a metastasis of a definite pathological diagnosis without a detectable primary site.

**Patients and methods:**

Patients diagnosed between 2004 and 2014 were identified from the Surveillance Epidemiology and End Results (SEER) database. The characteristics of type 2 CUP from different sources were analyzed. For each source of type 2 CUP, tumors of the corresponding T_n_N_0‐X_M_1_ stage were used as controls.

**Results:**

A total of 8505 patients with type 2 CUP were included in this analysis. Type 2 CUP shows an increasing trend, while type 1 shows the opposite. Type 2 CUPs have significant differences with stage IV of the same pathological primary lesion. Many characteristics influenced the prognosis of type 2 CUP patients, including marital status, age, race, sex, registration time, lymph node metastasis, surgery, chemotherapy, and radiation.

**Conclusion:**

Our study suggests that identifying the source of metastasis is the key to the selection of treatment and the determination of the prognosis for CUP.

## Graphical abstract

The number of type 2 cancer of unknown primary site (CUP) (metastatic cancer that the tissue or organ source can be determined but not the primary tumor, T_0_N_0‐X_M_1_) is increasing. Type 2 CUP (T_0_N_0‐X_M_1_) is a special cancer status, whose clinical characteristics and prognosis are significantly different from the classical CUP (type 1 CUP) and corresponding T_n_N_0‐X_M_1_. Identifying the source of metastatic focus is the key to the selection of treatment and prognosis for CUP.

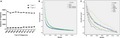

## 1 INTRODUCTION

Cancer of unknown primary site (CUP) is the seventh or eighth most common malignancy in various parts of the developed world, and it accounts for 2%‐5% of all malignancies and is the fourth most common cause of cancer‐related death.^1^, ^2^ A diagnosis of CUP should be limited to patients with histological confirmation of metastatic cancer, in whom a standard diagnostic approach does not reveal a primary tumor.^2^ According to the definition, there are still two states. One is that the tissue or organ source cannot be determined by pathological examination of metastatic carcinoma (mostly through autopsy); therefore, there is no primary tumor that can be located by clinical manifestation or imaging examination (Type 1). The other is that the tissue or organ source can be determined by pathological examination of the metastatic carcinoma, although no primary tumor can be detected by clinical manifestation or imaging examination, indicating that the T stage should be defined as T_0_ (Type 2). For the sake of differentiation, we classify them as type 1 and type 2, respectively. With the continuous updating and development of diagnostic methods (including pathological diagnosis, imaging methods and laboratory examinations), Type 1 is expected to be gradually overcome. It can be seen from the definition of CUP that T_0_N_0‐X_M_1_ is type 2 CUP, and the number of patients with this CUP will gradually rise with the continuous improvement in diagnostic and pathological technology and become the predominant type of CUP. A large‐scale population‐based study investigating the clinical characteristics of type 1 has been reported recently^3^; however, no report has yet involved type 2 CUP. In the current study, we used the surveillance epidemiology and end results (SEER) database to investigate the clinical presentation of patients with pathologically confirmed metastasis but no identified primary cancer sites (type 2 CUP) in a large population‐based study. The aim of the present study was to explore the clinical and prognostic features of type 2 CUP and the characteristics of metastasis from various histological origins.

## 2 METHODS

### 2.1 Patients

The data for this study were extracted from the SEER‐18 registry of the National Cancer Institute. The database is publicly available, and we retrieved the data using SEER*Stat Software Version 8.3.4. Because the SEER database contains deidentified data, this study was exempted from institutional review board oversight. We identified patients diagnosed between 1 January 2004, and 31 December 2014. Patients with type 1 CUP were defined as those patients for whom the primary site was classified as “unknown primary site” (ICD‐O‐3 code 80.9). T_0_N_0‐X_M_1_, which is type 2 CUP, was defined as American Joint Committee on Cancer (AJCC) stage (7th edition) ‘T_0_’ and ‘M_1_’ in the SEER database. For comparison, T_n_N_0‐X_M_1_ patients were defined as those with stages of “T_n_” and “M_1_” in the SEER database. Patients with unknown survival data or unknown treatment information were excluded. The characteristics of type 2 CUP from different sources were analyzed. For each source of type 2 CUP, tumors with corresponding T_n_N_0‐X_M_1_ stages were used as controls.

### 2.2 Data collection

The following demographic information for each patient was collected: age at diagnosis, year of diagnosis, sex, primary site of tumor, T stage, N stage, M stage, surgical resection of the primary site (yes or no), chemotherapy, marital status, survival months, and vital status. Information on systemic treatment was not provided in the SEER database.

### 2.3 Statistical analysis

The primary endpoint of this study was overall survival (OS). A chi‐square test was utilized to compare the differences in clinical and demographic features between different patient groups. OS was examined using the Kaplan‐Meier method with the log‐rank test. Multivariable survival analyses of OS were conducted using the Cox proportional hazards model. *P* < .05 was considered statistically significant. All statistical analyses were performed using IBM SPSS Statistics 22.0 (IBM).

## 3 RESULTS

### 3.1 Differences between type 1 and type 2 CUP

**Figure 1:**
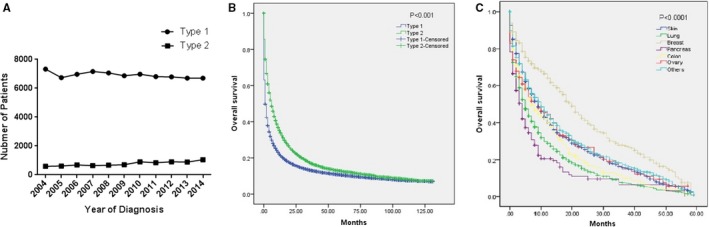
A, The curves of the number of cancer of unknown primary sites (CUPs) from 2004 to 2014. Type 2 CUP shows an annual increasing trend, while type 1 shows the opposite trend. B, Survival curves of CUPs; the prognosis of type 2 CUP is better than that of type 1. C, Survival analysis of the most common source of type 2 CUP. The prognoses of type 2 CUPs differed significantly based on the source

A total of 76 104 patients with type 1 CUP and 8505 with type 2 CUP were included in the current analysis (Table 1). The number of type 2 CUP patients is increasing, while the number of type 1 CUP patients shows the opposite trend (Figure 1A). Compared with patients with type 1, more patients in the type 2 group were married, older and male and underwent more treatment (surgery, radiation and chemotherapy, all *P* < .001, Table 1). Overall, the prognosis of type 2 CUP is better than that of type 1, and the OS curves are shown in Figure 1B. The median survival time was 1 month (95% CI = 0.97‐1.04) for type 1 CUP and 6 months (95% CI = 5.67‐6.33) for type 2 (*P* < .001).

**Table 1:** Baseline characteristics of cancer of unknown primary patients included in the analysis

Characteristics		Type 1		Type 2		*P* value
		(N = 76 104)	(%)	(N = 8505)	(%)	
Marital status	Married	32 711	43	4632	54.5	<.001
	Others	43 393	57	3873	45.5	
Age	<65	22 751	29.9	3699	43.5	<.001
	≥65	53 353	70.1	4806	56.5	
Gender	Male	37 362	49.1	4688	55.1	<.001
	Female	38 742	50.9	3817	44.9	
Surgery	Yes	6151	8.1	2218	26.1	<.001
	No	69 953	91.9	6287	73.9	
Radiation	Yes	10 348	13.6	2621	30.8	<.001
	No	65 756	86.4	5884	69.2	
Chemotherapy	Yes	14 017	18.4	3502	41.2	<.001
	No	62 087	81.6	5003	58.8	

### 3.2 Characteristics of type 2 CUP

The SEER database collected data on metastasis sites since 2010, and the analysis of type 2 CUP from 2010 to 2014 indicated that among the 4597 patients, liver metastasis occurred in 1368 patients (29.8%), lung metastasis occurred in 1343 (29.2%), bone metastasis occurred in 1271 (27.6%), and brain metastasis occurred in 991 (21.6%). The survival analysis revealed that undergoing surgical resection (HR = 1.985, 95% CI = 1.868‐2.109) and radiation (HR = 1.511, 95% CI = 1.435‐1.59) was associated with a better OS, and the factors associated with a poor OS included a single marital status, age ≥ 65 years, male sex, advanced N stage and earlier registration date (2004‐2008) (all *P* < .001, Table 2). Due to the heterogeneity of the type 2 CUP histological sources, multivariate analysis was not performed.

**Table 2:** Analysis of overall survival (OS) in type 2 cancer of unknown primary site

Parameter		Number	Univariate analysis	
			HR (95% CI)	*P* value
Marital status	Married	4632	0.862 (0.822‐0.905)	<.001
	Others	3873	1 (Referent)	
Age (y)	＜ 65	3699	0.682 (0.649‐0.716)	<.001
	≥ 65	4806	1 (Referent)	
Race	White	7480	0.985 (0.915‐1.061)	.695
	Others	1025	1 (Referent)	
Gender	Male	4688	1.126 (1.073‐1.182)	<.001
	Female	3817	1 (Referent)	
Year of diagnosis	2004‐2008	3208	0.929 (0.885‐0.976)	.003
	2009‐2014	5297	1 (Referent)	
N stage	N0	3548	1 (Referent)	
	Nn	2407	1.047 (0.987‐1.11)	.129
	NX	2550	1.213 (1.147‐1.284)	<.001
Surgery	No	6287	1.889 (1.782‐2.001)	<.001
	Yes	2218	1 (Referent)	
Radiation	No	5944	1.41 (1.343‐1.481)	<.001
	Yes	2561	1 (Referent)	
Chemotherapy	No	5003	1.06 (1.006‐1.116)	.029
	Yes	3502	1 (Referent)	

The most common sources of type 2 CUPs are the skin, lung, breast, pancreas, colon and ovary (Table 3). Since the most common sites of CUP2 are, in order, liver, lung, bone and brain, we further list the common pathologically confirmed primary sources for CUP2 with metastatic sites of the four organs respectively (Tables 4, 5, 6, 7), which also indicated that skin or lung are the commonest source for CUP2. The survival analysis suggested that the prognoses of type 2 CUP with different sources were significantly different. For example, among the six most common types of tumors, the one with the best prognosis and the one with the worst prognosis were breast cancer and pancreatic cancer, respectively (Figure 1C). Therefore, the characteristics of type 2 CUP with different sources were analyzed as follows. For each source of type 2 CUP, tumors with T_n_N_0‐X_M_1_ stages from the same source were used as controls.

**Table 3:** The list of common pathologically confirmed primary source of type 2 cancer of unknown primary site (CUP)

	Number	%
Source of CUP	8505	100
C44.9‐Skin, NOS	2972	34.9
C34.9‐Lung, NOS	1848	21.7
C50.9‐Breast, NOS	733	8.6
C25.9‐Pancreas, NOS	563	6.6
C18.9‐Colon, NOS	469	5.5
C56.9‐Ovary	288	3.4
C61.9‐Prostate gland	228	2.7
C64.9‐Kidney, NOS	144	1.7
C62.9‐Testis, NOS	117	1.4
C24.9‐Biliary tract, NOS	104	1.2
C16.9‐Stomach, NOS	71	0.8
Others	968	11.6

**Table 4:** The list of common pathologically confirmed primary source of type 2 cancer of unknown primary site (CUP) in liver

	Number	%
Source of CUP	1368	100
C44.9‐Skin, NOS	386	28.2
C34.9‐Lung, NOS	245	17.9
C25.9‐Pancreas, NOS	181	13.2
C18.9‐Colon, NOS	175	12.8
C50.9‐Breast, NOS	79	5.8
C24.9‐Biliary tract, NOS	48	3.5
C56.9‐Ovary	28	2
C24.0‐Extrahepatic bile duct	24	1.8
C16.9‐Stomach, NOS	17	1.2
C19.9‐Rectosigmoid junction	15	1.1
C23.9‐Gallbladder	15	1.1
Others	155	11.3

**Table 5:** The list of common pathologically confirmed primary source of type 2 cancer of unknown primary site (CUP) in lung

	Number	%
Source of CUP	1343	100
C44.9‐Skin, NOS	783	58.3
C18.9‐Colon, NOS	80	6
C50.9‐Breast, NOS	81	6
C34.9‐Lung, NOS	66	4.9
C25.9‐Pancreas, NOS	62	4.6
C56.9‐Ovary	32	2.4
C64.9‐Kidney, NOS	31	2.3
C62.9‐Testis, NOS	25	1.9
C24.9‐Biliary tract, NOS	18	1.3
C61.9‐Prostate gland	17	1.3
Others	148	11

**Table 6:** The list of common pathologically confirmed primary source of type 2 cancer of unknown primary site (CUP) in bone

	Number	%
Source of CUP	1271	100
C34.9‐Lung, NOS	331	26
C44.9‐Skin, NOS	301	23.7
C50.9‐Breast, NOS	238	18.7
C61.9‐Prostate gland	127	10
C64.9‐Kidney, NOS	47	3.7
C18.9‐Colon, NOS	34	2.7
C25.9‐Pancreas, NOS	34	2.7
C22.0‐Liver	16	1.3
C67.9‐Bladder, NOS	13	1
Others	130	10.2

**Table 7:** The list of common pathologically confirmed primary source of type 2 cancer of unknown primary site (CUP) in brain

	Number	%
Source of CUP	991	100
C44.9‐Skin, NOS	634	64
C34.9‐Lung, NOS	259	26.1
C50.9‐Breast, NOS	32	3.2
C18.9‐Colon, NOS	6	0.6
C25.9‐Pancreas, NOS	5	0.5
C34.1‐Upper lobe, lung	4	0.4
C34.3‐Lower lobe, lung	4	0.4
C44.6‐Skin of upper limb and shoulder	4	0.4
C61.9‐Prostate gland	4	0.4
C64.9‐Kidney, NOS	4	0.4
Others	35	3.5

### 3.3 Skin cancer

Compared with patients with T_n_N_0‐X_M_1_, skin cancer patients in the type 2 CUP group (T_0_N_0‐X_M_1_) were more often married and white, with lower N stages and more opportunities for surgery and radiation but fewer for chemotherapy (all *P* < .001, Table S1). Patients with type 2 CUP with skin cancer as the source also had a worse prognosis than the T_n_N_0‐X_M_1_ controls (Figure S1). Multivariate Cox regression analysis revealed that undergoing surgical resection (HR = 2.218, 95% CI = 2.027‐2.426) and chemotherapy (HR = 1.241, 95% CI = 1.4134‐1.357) was associated with a better OS, and other factors associated with poor OS included a single marital status, age ≥ 65 years, male sex and earlier registration date (2004‐2008) (Table S2).

### 3.4 Lung cancer

Lung cancer patients in the T_0_N_0‐X_M_1_ group (type 2 CUP) were more often married, younger, and white, and they had a lower N stage and more opportunities for treatment (surgery, radiation and chemotherapy, *P* < .001) when compared with patients in the T_n_N_0‐X_M_1_ group (Table S3). Patients in this type 2 CUP group also had a better prognosis (Figure S2A), including after surgical treatment (Figure S2B), compared with the T_n_N_0‐X_M_1_ group. Multivariate Cox regression analysis revealed that undergoing surgical resection (HR = 1.802, 95% CI = 1.566‐2.073), radiation (HR = 1.293, 95% CI = 1.16‐1.442) and chemotherapy (HR = 2.399, 95% CI = 2.153‐2.674) was associated with a better OS, and the factors associated with poor OS included a single marital status, age ≥ 65 years, white race, male sex, advanced N stage and earlier registration date (2004‐2008) (all *P* < .001, Table S4).

### 3.5 Breast cancer

Similarly, breast cancer patients with T_0_N_0‐X_M_1_ (type 2 CUP) were more often married, older, and white, and they had a lower N stage and more opportunities for surgery but fewer for radiation and chemotherapy (*P* < .001, Table S5). Patients in this type 2 CUP group also had a better prognosis compared with the corresponding T_n_N_0‐X_M_1_ patients (Figure S3A), although the difference was not significant after surgical treatment (Figure S3B). Multivariate Cox regression analysis revealed that undergoing surgical resection (HR = 1.408, 95% CI = 1.087‐1.825) was associated with a better OS, and the factors associated with a poor OS included a single marital status and earlier registration date (2004‐2008) (*P* < .001, Table S6).

### 3.6 Pancreatic cancer

Pancreatic cancer is one of the most malignant cancers. Pancreatic cancer patients with T_0_N_0‐X_M_1_ (type 2 CUP) were more often white, with a lower N stage and more opportunities for surgery (*P* < .001) when compared with the corresponding patients with T_n_N_0‐X_M_1_ (Table S7). Unlike other cancers, patients with type 2 CUP sourced from pancreatic cancer showed no difference in prognosis compared with those in the corresponding T_n_N_0‐X_M_1_ group (Figure S4A). Moreover, this type of type 2 CUP may result in a worse prognosis after surgery than T_n_N_0‐X_M_1_ (Figure S4B). Multivariate Cox regression analysis indicated that undergoing chemotherapy (HR = 2.438, 95% CI = 2.013‐2.954) was associated with a better OS, and the factors associated with a poor OS included age ≥ 65 years and male sex (all *P* < .001, Table S8).

### 3.7 Colon cancer

Colon cancer is the most common gastrointestinal malignancy; colon cancer patients with type 2 CUP were more often older, with a greater chance of being diagnosed recently (2009‐2014), a lower N stage and fewer opportunities for radiation (*P* < .001) when compared with the patients in the corresponding T_n_N_0‐X_M_1_ group (Table S9). Patients with this type of type 2 CUP also had a better prognosis than the patients in the corresponding T_n_N_0‐X_M_1_ group (Figure S5A), although that difference was no longer significant after surgical treatment (Figure S5B). Multivariate Cox regression analysis indicated that undergoing surgical resection (HR = 2.985, 95% CI = 2.118‐4.206) and chemotherapy (HR = 2.495, 95% CI = 2.003‐3.109) was associated with a better OS, and the factors associated with a poor OS included age ≥ 65 years and an earlier registration date (2004‐2008) (all *P* < .001, Table S10).

### 3.8 Ovarian cancer

Unlike in other cancers, compared with patients in the T_n_N_0‐X_M_1_ group, ovarian cancer patients in the T_0_N_0‐X_M_1_ group (type 2 CUP) were more often unmarried and older, were diagnosed in more recent years (2009‐2014), had a lower N stage and had more opportunities for radiation but fewer for surgery and chemotherapy (*P* < .001, Table S11). Patients in this type 2 CUP group had a worse prognosis than the patients in the corresponding T_n_N_0‐X_M_1_ group (Figure S6A), although the difference was not significant after surgical treatment (Figure S6B). Multivariate Cox regression analysis revealed that undergoing chemotherapy (HR = 3.101, 95% CI = 2.333‐1.123) but not surgical resection or radiation was associated with a better OS, and age ≥ 65 years was associated with a poor OS (all *P* < .001, Table S12).

### 3.9 Prostate cancer

Prostate cancer patients with type 2 CUP were older than those in the T_n_N_0‐X_M_1_ group and also had a greater chance of being diagnosed recently (2009‐2014), a lower N stage and more opportunities for surgical treatment (*P* < .001, Table S13). Patients with this type of type 2 CUP also had a worse prognosis than the patients in the corresponding T_n_N_0‐X_M_1_ group (Figure S7A), although the difference was not significant after surgical treatment (Figure S7B). Multivariate Cox regression analysis revealed that undergoing surgical resection (HR = 2.262, 95% CI = 1.21‐4.23) was associated with a better OS, and age ≥ 65 years was associated with a poor OS (*P* < .001, Table S14).

### 3.10 Kidney cancer

Compared with patients in the T_n_N_0‐X_M_1_ group, kidney cancer patients in the T_0_N_0‐X_M_1_ group (type 2 CUP) had a lower N stage, were diagnosed more recently (2009‐2014) and had more opportunities for surgery (*P* < .001, Table S15). Compared with T_n_N_0‐X_M_1_ patients, the type 2 CUP group had a worse prognosis (Figure S8A), even after surgery (Figure S8B). Multivariate Cox regression analysis demonstrated that age ≥ 65 years was associated with a poor OS (*P* < .001, Table S16).

### 3.11 Testicular cancer

Like most other patients with type 2 CUP, testicular cancer patients with type 2 CUP were more often married and older with advanced N stages and more opportunities for surgery and radiation (*P* < .001, Table S17). Patients with this type of type 2 CUP also had a worse prognosis than the patients in the corresponding T_n_N_0‐X_M_1_ group (Figure S9A), although the difference was not significant after surgery (Figure S9B). Multivariate Cox regression analysis revealed that undergoing surgical resection (HR = 2.856, 95% CI = 1.211‐6.736) was associated with a better OS (*P* < .001, Table S18).

### 3.12 Biliary tract cancer

Compared with patients in the T_n_N_0‐X_M_1_ group, biliary tract cancer patients in the T_0_N_0‐X_M_1_ group (type 2 CUP) had a lower N stage (Table S19). The prognosis was similar between the two groups, regardless of surgery (Figure S10A,B). Multivariate Cox regression analysis showed that undergoing chemotherapy (HR = 3.123, 95% CI = 1.882‐5.182) was associated with a better OS (*P* < .001, Table S20).

### 3.13 Stomach cancer

Stomach cancer patients with type 2 CUP had a lower N stage than those with T_n_N_0‐X_M_1_ (Table S21), although the prognosis was similar between the two groups, regardless of whether surgery was performed (Figure S11A,B). Multivariate Cox regression analysis indicated that undergoing chemotherapy (HR = 1.983, 95% CI = 1.112‐3.539) was associated with a better OS (*P* < .001, Table S22).

## 4 DISCUSSION

CUP is recognized as being a heterogeneous entity with a wide variety of presentations and is usually characterized by aggressive or unpredictable behavior and a poor prognosis.^4^ Our study describes a special type of CUP (type 2), in which the pathologically confirmed metastasis can be identified but not the primary cancer site. Our results show an increasing trend in the incidence of type 2 CUP and a decreasing trend in the incidence of classic CUP (type 1). The inability to detect the primary sites of type 1 CUP may have been due to the limitations of the pathological techniques at the time. It has been indicated that the primary anatomical sites in patients with CUP are identified in approximately 75% of postmortem examinations, and most are less than 1 cm in size.^5^ With the development of pathological technology, some metastases that used to be classified as type 1 CUP are now classified as type 2. Therefore, the number of type 1 CUPs has decreased, and the number of type 2 CUPs has increased. There are many similarities between these two types of CUP, and the most common sites of metastasis in type 2 CUP are, in order, the liver, lung, bone and brain, which is similar to the findings in type 1 CUP^2^; however, our study shows that patients with type 2 CUP have many differences compared with patients with type 1 CUP, such as marital status, sex, age and the frequency with which they undergo treatment, and it is worth emphasizing that patients with type 2 CUP have a much better prognosis than those with type 1. Therefore, it is important to detect the characteristics of type 2 CUP. The primary sites of type 2 CUP, in order from most to least common, are the skin, lung, breast, pancreas, colon, and ovary. Despite some differences, the majority of metastatic lesions of type 2 CUP in the liver and lungs were originated from the skin and lungs. Although our analysis shows that many clinical factors may influence the prognosis of type 2 CUP, including treatment methods, such as surgery and chemotherapy, other factors such as marital status, age, sex, N stage, and registration time are also involved. However, there are considerable differences among the prognoses of these patients; for example, type 2 CUP originating from breast cancer has the best prognosis, while that originating from the pancreas has the worst prognosis. These results are in agreement with those of a previous study.^6^ Therefore, to detect the specific role of each source cancer of type 2 CUP, the corresponding T_n_N_0‐X_M_1_ group was used as the control.

Patients with type 2 CUPs originating from the skin, lung, breast, and testis have a greater chance of being married, which may be because married people are concerned about their spouses. Tn stage cancer with detectable primary sites will be treated at an early stage. Only those without a primary tumor may have the chance to develop an advanced stage with metastasis. For the same reason, married persons with type 2 CUPs originating in the skin, lung, and breast have a better prognosis than unmarried persons, and such results are in agreement with those of previous studies.^7^, ^8^, ^9^, ^10^, ^11^ However, compared with the corresponding T_n_N_0‐X_M_1_ group, patients with type 2 CUP originating from the ovary tend to be unmarried, which may be explained by the influence of the estrogen level.^12^

Younger individuals more often develop metastases with detectable primary sites (T_n_N_0‐X_M_1_) originating from the breast, colon, ovary, prostate and testis, which may be explained by the relatively healthy condition and strong immune system of these patients prohibiting early metastasis. This can also explain the better prognosis of younger patients with type 2 CUP originating from the skin, lung, pancreas, colon, ovary, prostate gland and kidney. However, with regard to type 2 CUP originating from the lung cancer, older patients tend to develop metastasis without a primary site, which may be because the older patients are more likely to be undergoing routine clinical examinations. It has been suggested that a routine clinical examination will help to diagnose cancer at an early and treatable stage.^13^ Therefore, cancer with identifiable primary sites will receive early treatment, and only those without primary sites will have the chance to develop metastases.

For type 2 CUP originating from the skin, lung, breast and pancreas, white individuals more often develop metastases without primary sites, which may be due to the greater attention paid to their health status, and Tn stage tumors are less likely to develop metastases. The survival analysis indicated that among patients with type 2 CUP originating from the lung, white individuals tend to have a better prognosis than other individuals, which is in agreement with the findings of a previous study.^14^ Reviews suggest that the reasons for the wide racial disparities in lung cancer survival are complex and multifactorial, with contributions from treatment‐related factors, such as physician‐patient encounters and decision‐making, and barriers to access to high‐quality care, such as lower patient income or insurance coverage limits.^15^, ^16^, ^17^

A sex‐based difference was only detected in lung cancer, and male patients were more likely to develop type 2 CUP. This may be due to the more widespread smoking habit among men. The survival analysis showed that among patients with type 2 CUP originating from the skin and lung, male patients had a worse prognosis, which may be explained by less attention paid to their health. Therefore, it is reasonable that females have a better prognosis than males. Female sex has already proved to be a favorable prognostic factor for lung cancer.^18^ There are limited reports about the effect of sex on prognosis in patients with skin cancer; however, a strong protective effect of female sex against mortality has been confirmed in patients with skin melanoma.^19^

The number of patients with later registration dates who were diagnosed with type 2 CUP originating from the lung, colon, ovary, prostate gland and kidney was much higher than in the corresponding T_n_N_0‐X_M_1_ group, which may be due to the development of diagnostic technology. This can also explain why patients with type 2 CUP originating from the skin and lung with later registration dates also have a better prognosis than before. Our results are consistent with those of other reports on lung cancer,^14^ which may be due to advances in medical treatment.

Most type 2 CUPs have a lower N stage, which was true of almost all the 11 cancers we studied (except prostate cancer). Such results may be due to the stronger invasion and metastatic potential of type 2 CUPs, which may develop metastases at a very early T stage. Negative lymph node metastasis indicates a better prognosis of type 2 CUP originating from the lung. This result is consistent with the results of previous studies.^20^, ^21^

Surgical treatment of metastases was performed more for type 2 CUPs originating from the skin, lung, pancreas, prostate gland, kidney, testis and stomach, which may be because such treatment provides a greater chance to obtain a radical excision of the cancer after the resection of the metastases. However, this situation was the opposite in ovarian cancer, which may be because the control of the estrogen level has more therapeutic value than surgery for metastases.^22^, ^23^ Surgical treatment of metastases is an effective measure for type 2 CUP originating from the skin, lung, breast and testis. This is because the resection of metastases means a radical removal of type 2 CUP. Therefore, for type 2 CUPs originating from the skin, lung, breast and testis, surgery is highly recommended. The surgical resection of metastases may also be effective for some other type 2 CUPs. Our study did not reach such a conclusion, possibly because of the small number of patients. Interestingly, type 2 CUPs originating from the lung and breast have a better prognosis than the corresponding T_n_N_0‐X_M_1_ groups; however, type 2 CUPs originating from the skin, colon, ovary, prostate gland, kidney and testis have worse prognoses than their corresponding control groups. Moreover, type 2 CUP originating from the lung has a better prognosis than the corresponding T_n_N_0‐X_M_1_ group after the surgical treatment of the metastases_._ However, type 2 CUP originating from the pancreas and kidney had a worse prognosis than the corresponding controls. This difference in prognosis agrees with the special properties of the different primary sites.^6^

Radiation treatment of metastases was performed more for type 2 CUP originating from the skin, lung, colon, ovary and testis but less for that originating from the breast. This difference may be because metastatic breast cancer is more sensitive to endocrine therapy, chemotherapy or molecularly targeted drug therapy.^24^, ^25^ The survival analysis indicated that radiation only had a beneficial effect for those with metastases from lung cancer, and radiation is one of the main treatment methods for lung cancer.^26^ However, radiation may deteriorate the condition of skin cancer, which is not sensitive to radiotherapy. Additionally, UV radiation is a carcinogen known to play a role in the development of non‐melanoma and melanoma skin cancers.^27^

Chemotherapy is adopted more for metastatic cancer with detectable primary sites originating from the skin, breast and ovary but less for those originating from the lung. This may be because chemotherapy is an effective systemic therapy for those cancers 28, 29, 30 but it is less often selected as a treatment for lung cancer because it is hard to identify primary and metastatic lung lesions. Our results show that chemotherapy is an effective treatment measure for metastases originating from the skin, lung, pancreas, colon, ovary, biliary tract and stomach. This result can be easily explained by the sensitivity of the primary cancer to chemotherapy.

Due to the lack of reports on type 2 CUP and the lack of studies on its mechanisms, the interpretation of many results of this study needs to be investigated in further studies. However, our study strongly suggests that identifying the source of metastatic focus is the key to the selection of treatment and a better prognosis.

In conclusion, our study suggests that identifying the source of metastatic focus is the key to the selection of treatment and improving the prognosis for patients with CUP. Type 2 CUPs have significant differences when compared with stage IV neoplasms of the same pathological primary lesion. Many characteristics influenced the prognosis of type 2 CUP patients, including marital status, age, race, sex, registration date, lymph node metastasis, surgery, chemotherapy, and radiation.

## CONFLICT OF INTEREST

None declared.

## AUTHOR CONTRIBUTIONS

Dr Deyu Li and Lianyuan Tao conceived and designed this study; Lianyuan Tao performed the data analysis; Lianyuan Tao, Deyu Li, Haibo Yu, Yadong Dong, Guanjing Tian and Zhiyuan Ren interpreted the data; and Deyu Li and Lianyuan Tao wrote the manuscript. All authors reviewed and accepted the final manuscript for submission.
