# Supplementary figures and images for: Metastases with definitive pathological diagnosis but no detectable primary tumor: A surveillance epidemiology and end results‐based study
Source: Cancer Med. 2019 Aug 13;8(13):5872–80. doi: 10.1002/cam4.2496 (PMC6792521; doi:10.1002/cam4.2496)

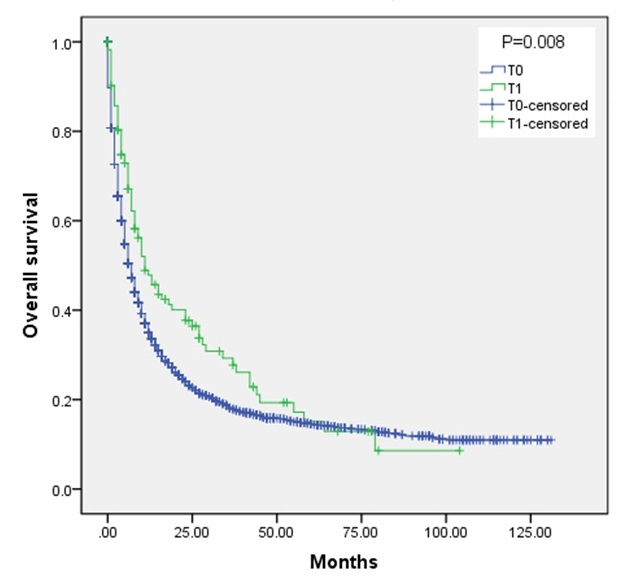

Supplement: Supplementary file 1 [file CAM4-8-5872-s001.tif]

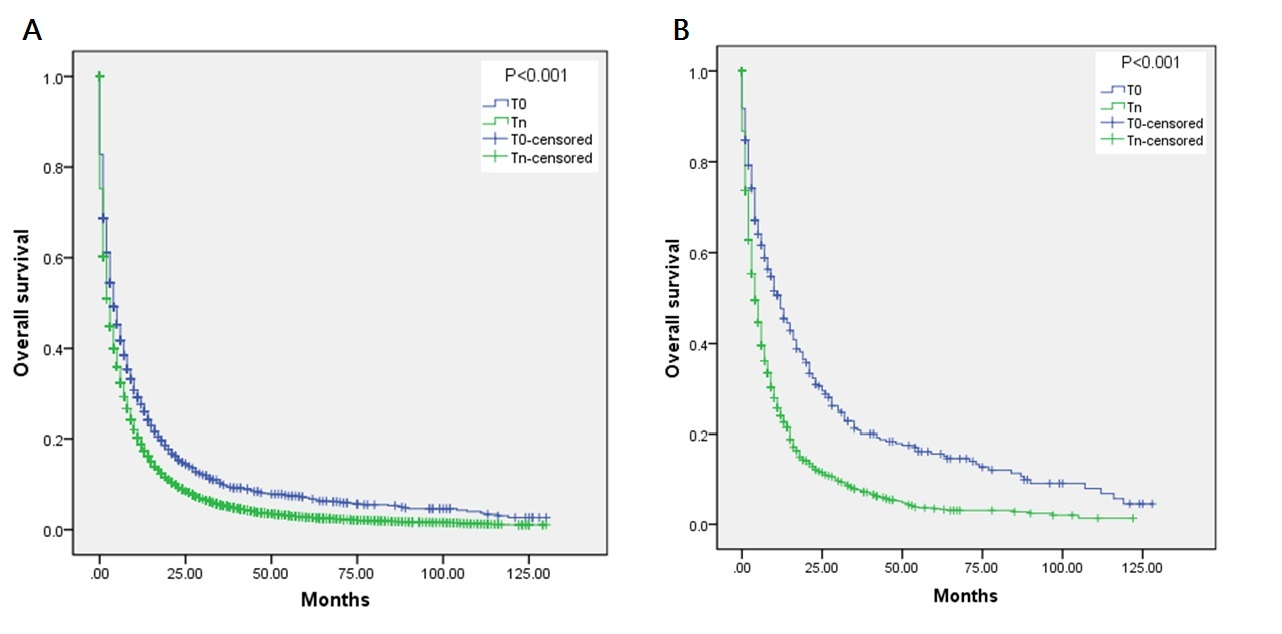

Supplement: Supplementary file 2 [file CAM4-8-5872-s002.tif]

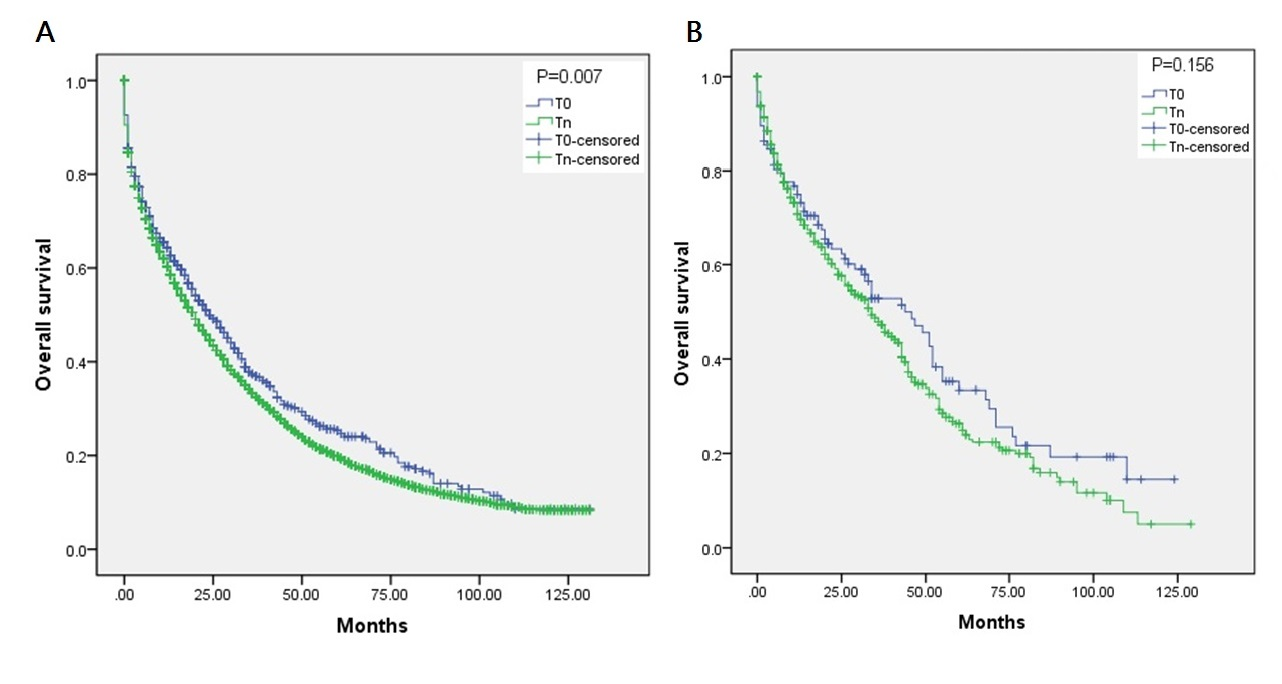

Supplement: Supplementary file 3 [file CAM4-8-5872-s003.tif]

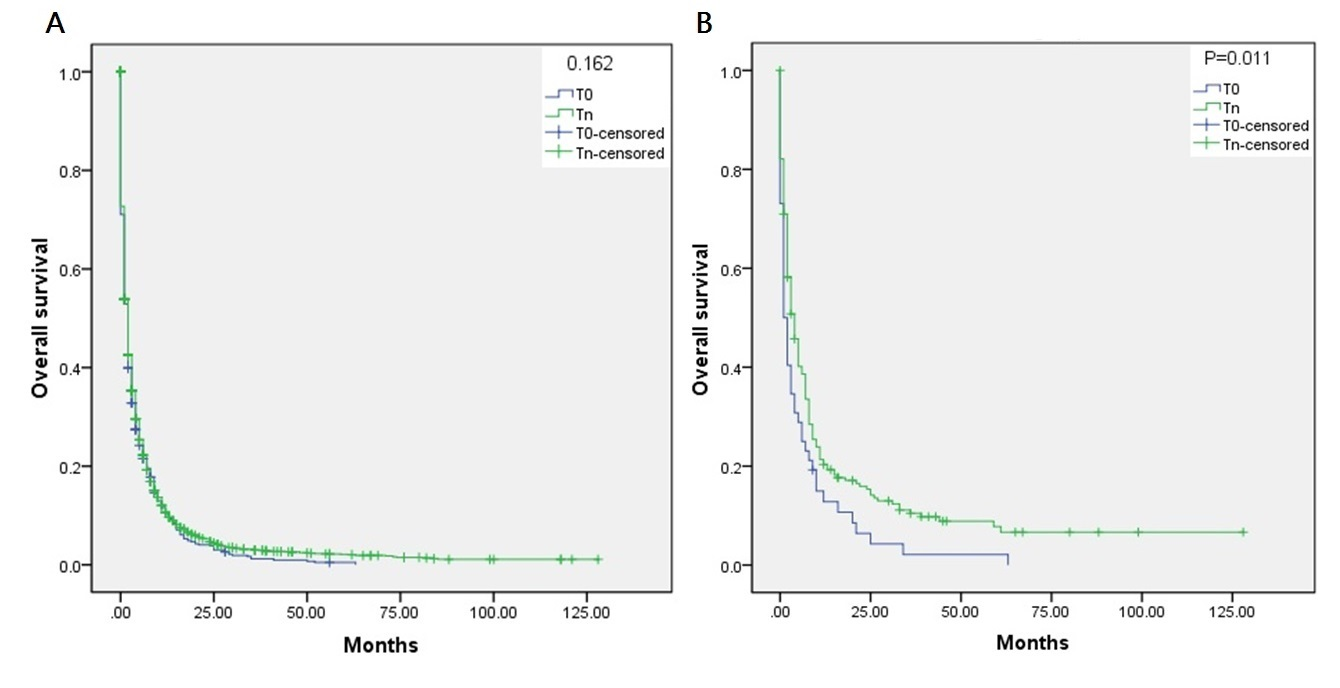

Supplement: Supplementary file 4 [file CAM4-8-5872-s004.tif]

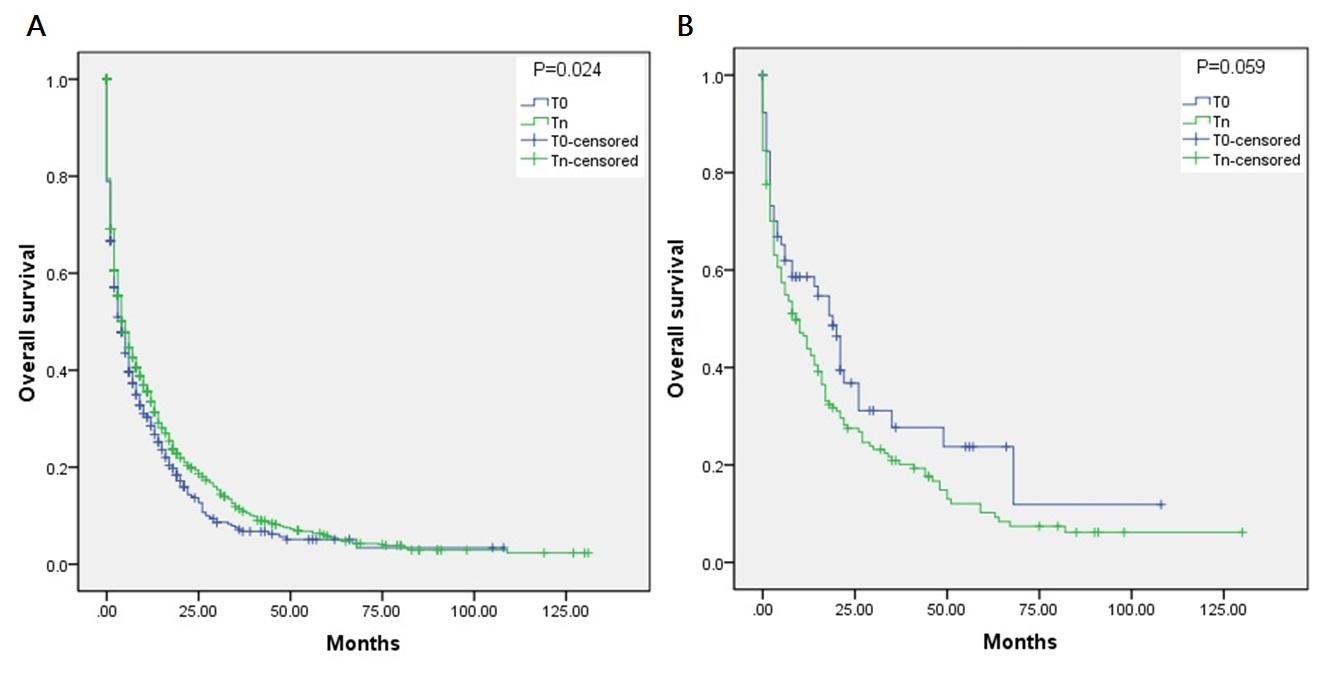

Supplement: Supplementary file 5 [file CAM4-8-5872-s005.tif]

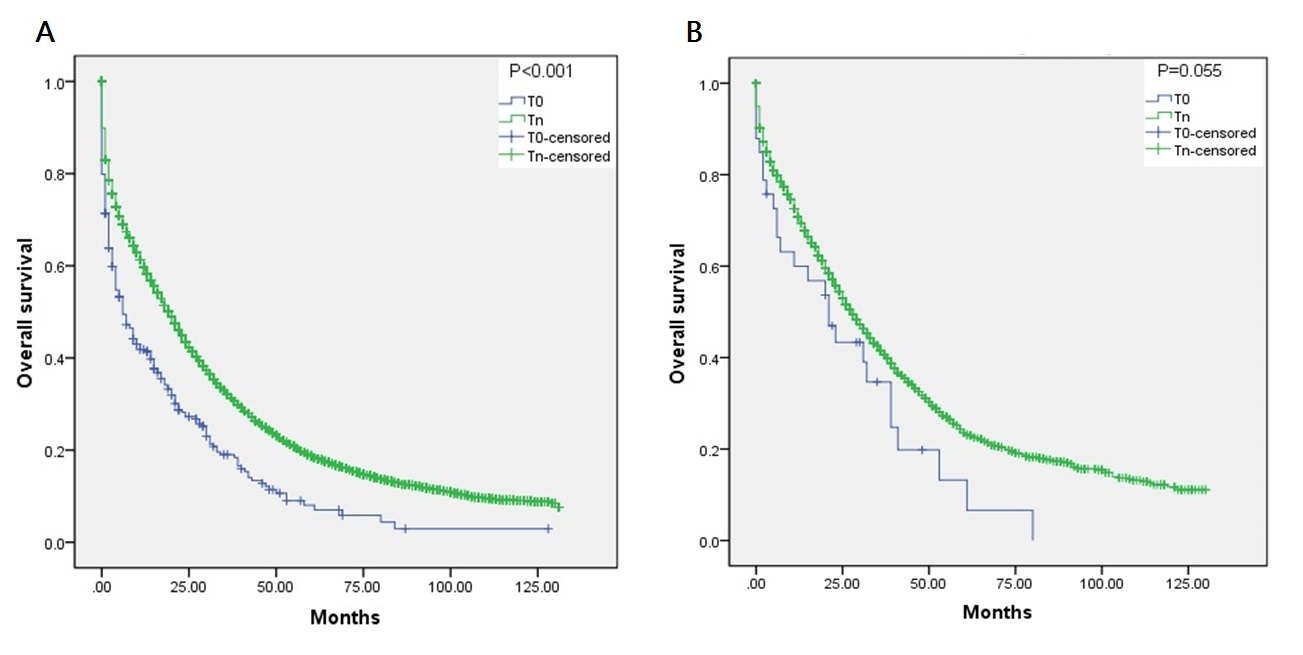

Supplement: Supplementary file 6 [file CAM4-8-5872-s006.tif]

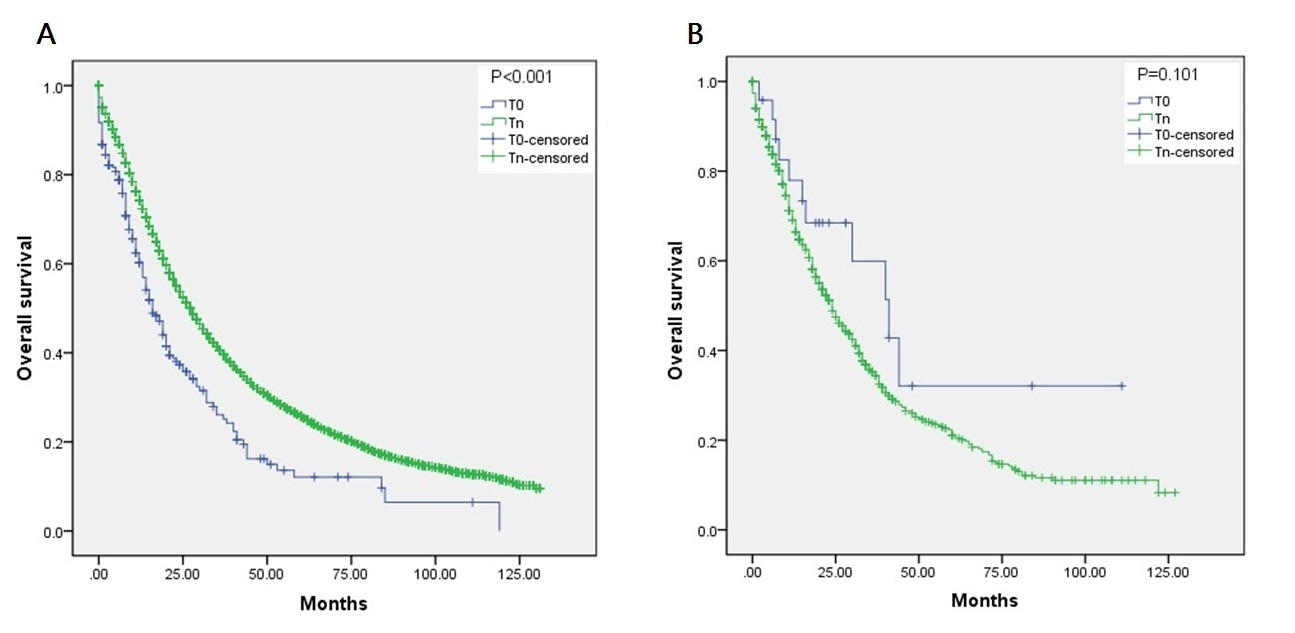

Supplement: Supplementary file 7 [file CAM4-8-5872-s007.tif]

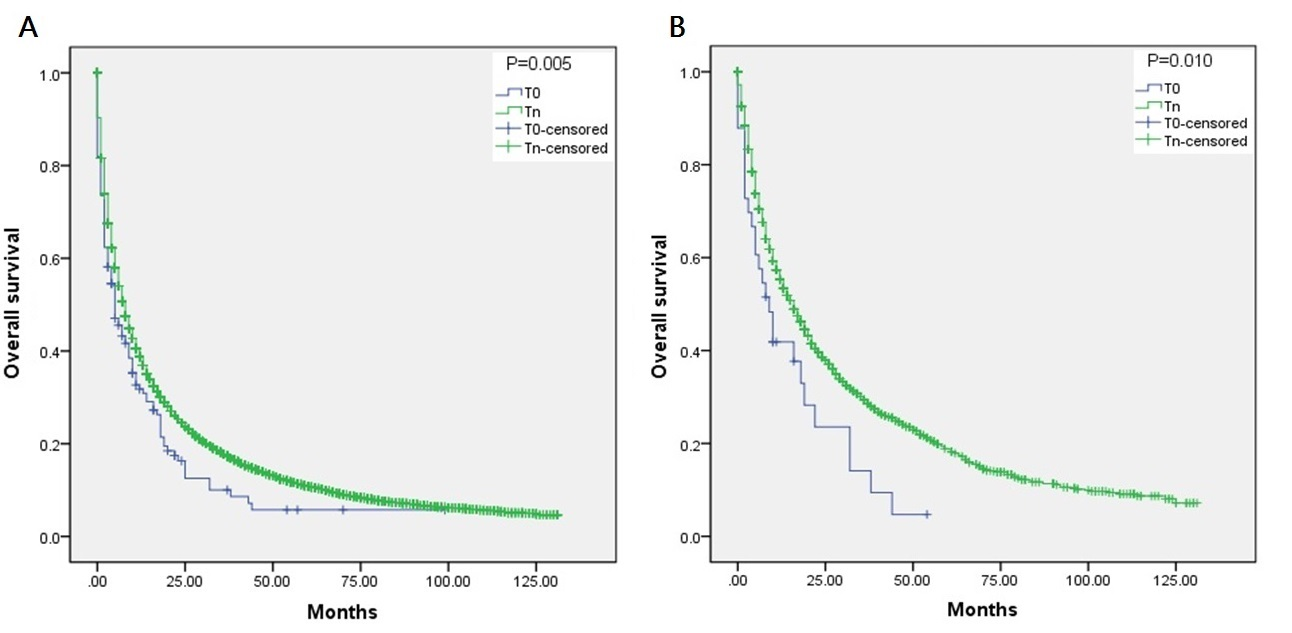

Supplement: Supplementary file 8 [file CAM4-8-5872-s008.tif]

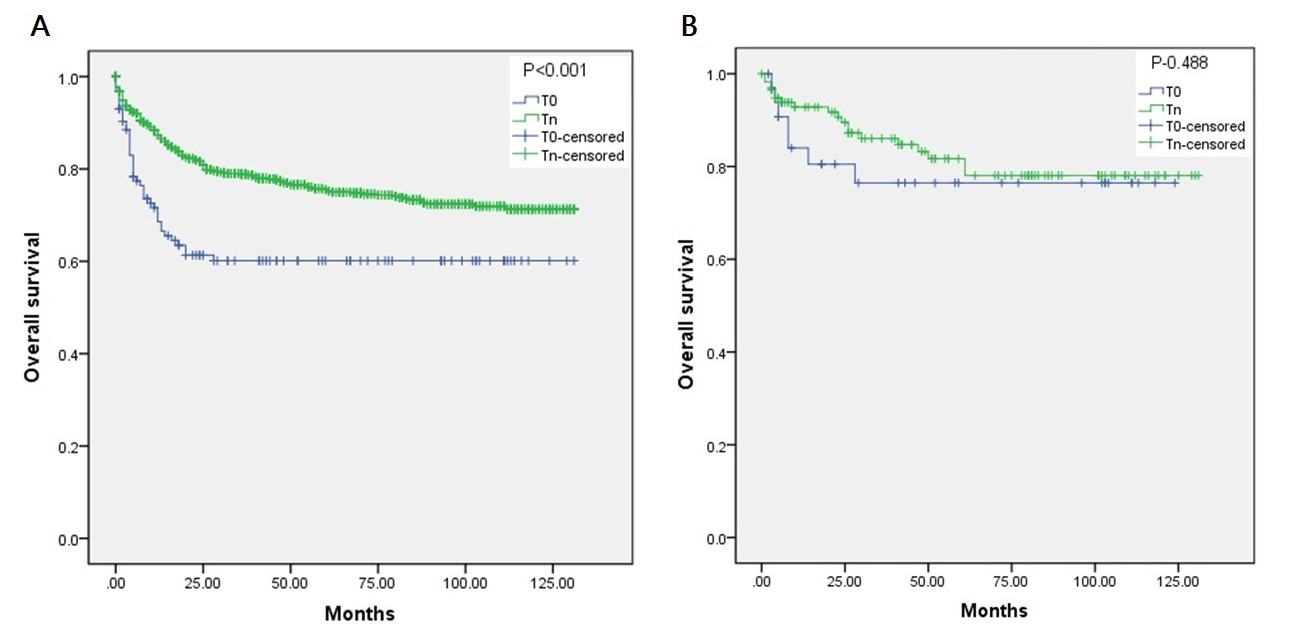

Supplement: Supplementary file 9 [file CAM4-8-5872-s009.tif]

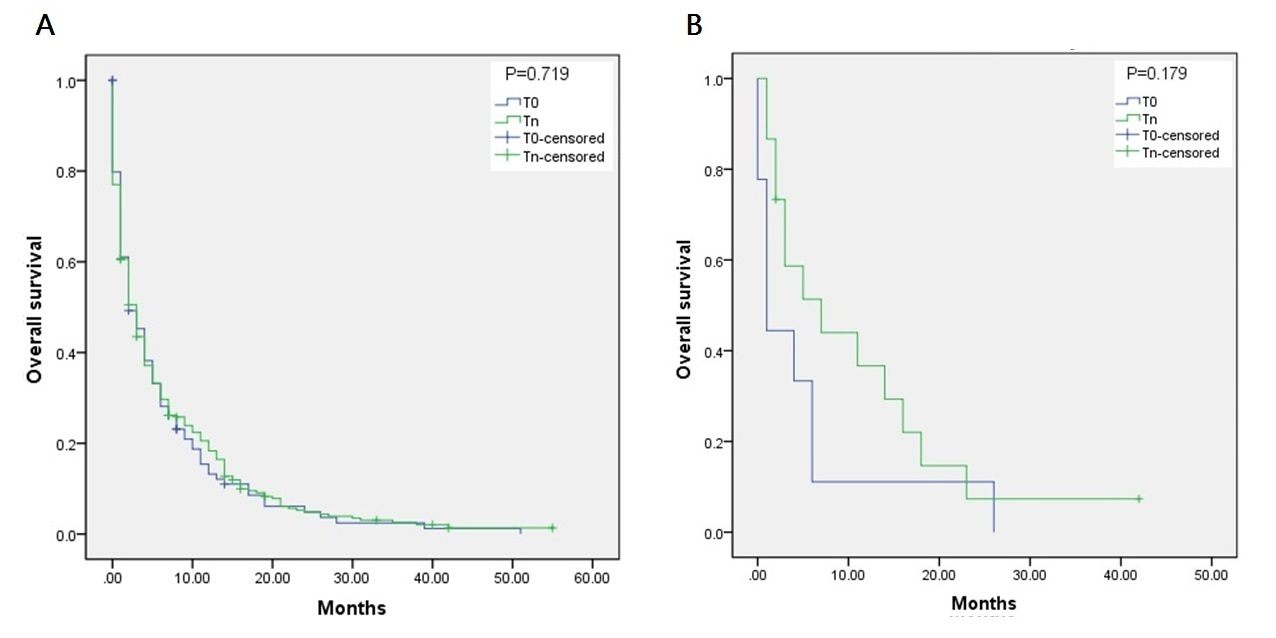

Supplement: Supplementary file 10 [file CAM4-8-5872-s010.tif]

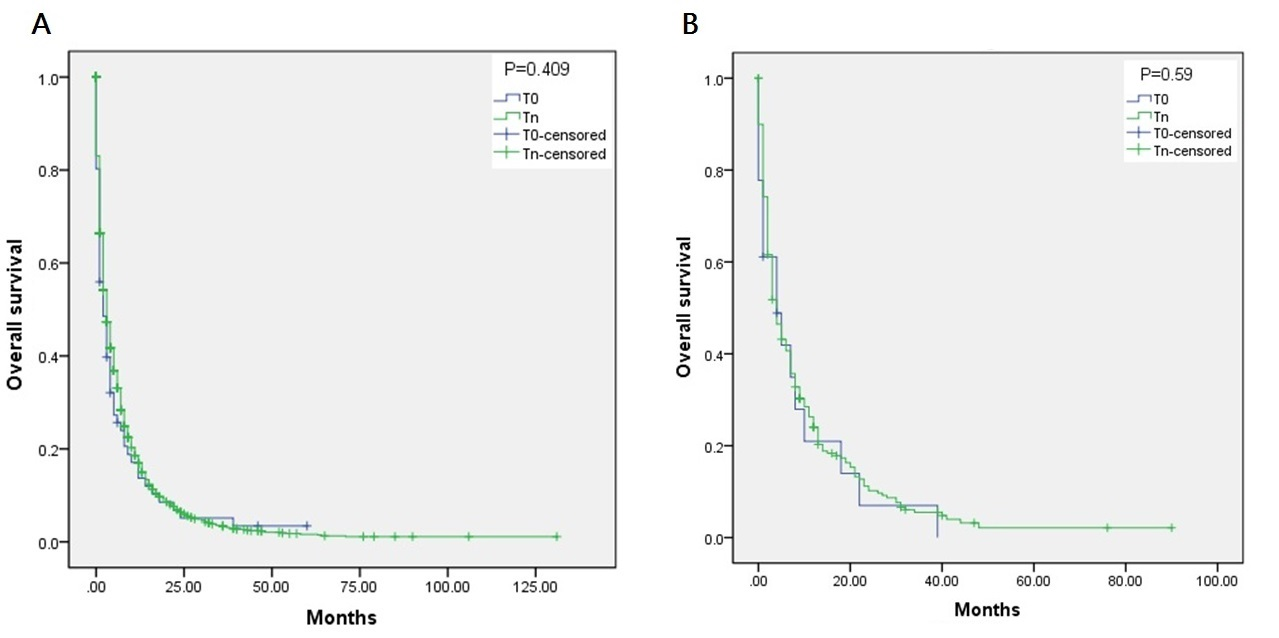

Supplement: Supplementary file 11 [file CAM4-8-5872-s011.tif]
