# Supplementary material for: Metastases with definitive pathological diagnosis but no detectable primary tumor: A surveillance epidemiology and end results‐based study
Source: Cancer Med. 2019 Aug 13;8(13):5872–80. doi: 10.1002/cam4.2496 (PMC6792521; doi:10.1002/cam4.2496)
Supplement: Supplementary file 12 [file CAM4-8-5872-s012.doc]

Table S1. Comparison of the clinical parameters of type 2 CUP and T_n_N_0-X_M_1_ in skin cancer patients .

|  |  | **Type 2** CUP |  | T_n_N_0-X_M_1_ |  | **P value** |
| --- | --- | --- | --- | --- | --- | --- |
| **Parameters** |  | 2972 | 100.00% | 114 | 100.00% |  |
| **Marital status** | **Married** | 1821 | 61.30% | 48 | 42.10% | <0.001 |
|  | Others | 1151 | 38.70% | 66 | 57.90% |  |
| **Age (years)** | ＜65 | 1440 | 48.50% | 52 | 45.60% | 0.568 |
|  | ≥65 | 1532 | 51.50% | 62 | 54.40% |  |
| **Race** | White | 2864 | 96.40% | 90 | 78.90% | <0.001 |
|  | Others | 108 | 3.60% | 24 | 21.10% |  |
| **Gender** | Male | 2035 | 68.50% | 75 | 65.80% | 0.539 |
|  | Female | 937 | 31.50% | 39 | 34.20% |  |
| **Year of diagnosis** | 2004-2008 | 1116 | 37.60% | 51 | 44.70% | 0.14 |
|  | 2009-2014 | 1856 | 62.40% | 63 | 55.30% |  |
| **N stage** | N0 | 1085 | 36.50% | 34 | 29.80% | <0.001 |
|  | Nn | 512 | 17.20% | 59 | 51.80% |  |
|  | NX | 1375 | 46.30% | 21 | 18.40% |  |
| **Surgery** | No | 1688 | 56.80% | 104 | 91.20% | <0.001 |
|  | Yes | 1284 | 43.20% | 10 | 8.80% |  |
| **Radiation** | No | 1764 | 59.40% | 99 | 86.80% | <0.001 |
|  | Yes | 1208 | 40.60% | 15 | 13.20% |  |
| **Chemotherapy** | No | 2005 | 67.50% | 56 | 49.10% | <0.001 |
|  | Yes | 967 | 32.50% | 58 | 50.90% |  |

Table S2. Analysis of overall survival (OS) in type 2 CUP in skin cancer patients .

| **Parameter** |  | | |  | |  | **Univariate analysis** | | | Multivariate analysis | | |
| --- | --- | --- | --- | --- | --- | --- | --- | --- | --- | --- | --- | --- |
|  | |  | Number | | % | | | HR(95% CI) | P value | HR(95% CI) | | P value |
| **Marital status** | | **Married** | 1821 | | 61.3 | | | 0.843(0.775-0.917) | <0.001 | 0.783(0.719-0.853) | | <0.001 |
|  | | Others | 1151 | | 38.7 | | | 1(Referent) |  | 1(Referent) | |  |
| **Age (years)** | | ＜65 | 1440 | | 48.5 | | | 0.778(0.717-0.844) | <0.001 | 0.804(0.739-0.875) | | <0.001 |
|  | | ≥65 | 1532 | | 51.5 | | | 1(Referent) |  | 1(Referent) | |  |
| **Race** | | White | 2864 | | 96.4 | | | 0.957(0.771-1.189) | 0.694 | 1.054(0.847-1.311) | | 0.48 |
|  | | Others | 108 | | 3.6 | | | 1(Referent) |  | 1(Referent) | |  |
| **Gender** | | Male | 2035 | | 68.5 | | | 1.175(1.075-1.285) | <0.001 | 1.237(1.129-1.356) | | <0.001 |
|  | | Female | 937 | | 31.5 | | | 1(Referent) |  | 1(Referent) | |  |
| **Year of diagnosis** | | 2004-2008 | 1116 | | 37.6 | | | 1.097(1.009-1.193) | 0.029 | 1.558(1.401-1.733) | | <0.001 |
|  | | 2009-2014 | 1856 | | 62.4 | | | 1(Referent) |  | 1(Referent) | |  |
| **N stage** | | N0 | 1085 | | 36.5 | | | 1(Referent) |  | 1(Referent) | |  |
|  | | Nn | 512 | | 17.2 | | | 1.035(0.915-1.171) | 0.582 | 0.947(0.836-1.072) | | 0.388 |
|  | | NX | 1375 | | 46.3 | | | 1.295(1.182-1.418) | <0.001 | 1.256(1.145-1.377) | | <0.001 |
| **Surgery** | | No | 1688 | | 56.8 | | | 2.237(2.052-2.438) | <0.001 | 2.218(2.027-2.426) | | <0.001 |
|  | | Yes | 1284 | | 43.2 | | | 1(Referent) |  | 1(Referent) | |  |
| **Radiation** | | No | 1764 | | 59.4 | | | 0.824(0.758-0.895) | <0.001 | 0.906(0.826-0.995) | <0.001 | |
|  | | Yes | 1208 | | 40.6 | | | 1(Referent) |  | 1(Referent) |  | |
| **Chemotherapy** | | No | 2005 | | 67.5 | | | 1.082(0.992-1.18) | 0.074 | 1.241(1.134-1.357) | <0.001 | |
|  | | Yes | 967 | | 32.5 | | | 1(Referent) |  | 1(Referent) |  | |

Table S3. Comparison of the clinical parameters of type 2 CUP and T_n_N_0-X_M_1_ in lung cancer patients.

| **Parameters** |  | | | **Type 2** CUP |  | T_n_N_0-X_M_1_ | |  | | **P value** | |
| --- | --- | --- | --- | --- | --- | --- | --- | --- | --- | --- | --- |
|  | |  | N=1848 | 100.00% | N=31321 | | 100.00% | |  | |  |
| **Marital status** | | **Married** | 960 | 51.90% | 14919 | | 47.60% | | <0.001 | |  |
|  | | Others | 888 | 48.10% | 16402 | | 52.40% | |  | |  |
| **Age (years)** | | ＜65 | 719 | 38.90% | 10865 | | 34.70% | | <0.001 | |  |
|  | | ≥65 | 1129 | 61.10% | 20456 | | 65.30% | |  | |  |
| **Race** | | White | 1594 | 86.30% | 25299 | | 80.80% | | <0.001 | |  |
|  | | Others | 254 | 13.70% | 6022 | | 19.20% | |  | |  |
| **Gender** | | Male | 1078 | 58.30% | 16902 | | 54.00% | | <0.001 | |  |
|  | | Female | 770 | 41.70% | 14419 | | 46.00% | |  | |  |
| **Year of diagnosis** | | 2004-2008 | 738 | 39.90% | 14020 | | 44.80% | | <0.001 | |  |
|  | | 2009-2014 | 1110 | 60.10% | 17301 | | 55.20% | |  | |  |
| **N stage** | | N0 | 554 | 30.00% | 6832 | | 21.80% | | <0.001 | |  |
|  | | Nn | 1031 | 55.80% | 20810 | | 66.40% | |  | |  |
|  | | NX | 263 | 14.20% | 3679 | | 11.70% | |  | |  |
| **Surgery** | | No | 1504 | 81.40% | 29953 | | 95.60% | | <0.001 | |  |
|  | | Yes | 344 | 18.60% | 1368 | | 4.40% | |  | |  |
| **Radiation** | | No | 1104 | 59.70% | 22118 | | 70.60% | | <0.001 | |  |
|  | | Yes | 744 | 40.30% | 9203 | | 29.40% | |  | |  |
| **Chemotherapy** | | No | 930 | 50.30% | 17662 | | 56.40% | | <0.001 | |  |
|  | | Yes | 918 | 49.70% | 13659 | | 43.60% | |  | |  |

Table S4. Analysis of overall survival (OS) in type 2 CUP in lung cancer patients.

| **Parameter** |  |  |  | **Univariate analysis** | | **Multivariate analysis** | |
| --- | --- | --- | --- | --- | --- | --- | --- |
|  |  | Number | % | HR(95% CI) | P value | HR(95% CI) | P value |
| **Marital status** | **Married** | **960** | **51.90%** | 0.863(0.843-0.882) | <0.001 | 0.897(0.81-0.993) | 0.035 |
|  | Others | 888 | 48.10% | 1(Referent) |  | 1(Referent) |  |
| **Age (years)** | ＜65 | 719 | 38.90% | 0.675(0.609-0.748) | <0.001 | 0.763(0.686-0.848) | <0.001 |
|  | ≥65 | 1129 | 61.10% | 1(Referent) |  | 1(Referent) |  |
| **Race** | White | 1594 | 86.30% | 1.34(1.154-1.557) | <0.001 | 1.353(1.163-1.573) | <0.001 |
|  | Others | 254 | 13.70% | 1(Referent) |  | 1(Referent) |  |
| **Gender** | Male | 1078 | 58.30% | 1.142(1.116-1.168) | <0.001 | 1.142(1.03-1.267) | <0.001 |
|  | Female | 770 | 41.70% | 1(Referent) |  | 1(Referent) |  |
| **Year of diagnosis** | 2004-2008 | 738 | 39.90% | 1.095(0.070-1.120) | <0.001 | 1.149(1.014-1.303) | <0.001 |
|  | 2009-2014 | 1110 | 60.10% | 1(Referent) |  | 1(Referent) |  |
| **N stage** | N0 | 554 | 30.00% | 1(Referent) |  | 1(Referent) |  |
|  | Nn | 1031 | 55.80% | 1.28 (1.244-1.316) | <0.001 | 1.541(1.497-1.586) | <0.001 |
|  | NX | 263 | 14.20% | 1.427(1.370-1.486) | <0.001 | 1.440(1.383-1.499) | <0.001 |
| **Surgery** | No | 1504 | 81.40% | 1.772(1.554-2.021) | <0.001 | 1.802(1.566-2.073) | <0.001 |
|  | Yes | 344 | 18.60% | 1(Referent) |  | 1(Referent) |  |
| **Radiation** | No | 1104 | 59.70% | 1.465(1.324-1.62) | <0.001 | 1.293(1.16-1.442) | <0.001 |
|  | Yes | 744 | 40.30% | 1(Referent) |  | 1(Referent) |  |
| **Chemotherapy** | No | 930 | 50.30% | 2.163(1.957-2.391) | <0.001 | 2.399(2.153-2.674) | <0.001 |
|  | Yes | 918 | 49.70% | 1(Referent) |  | 1(Referent) |  |

Table S5. Comparison of the clinical parameters of type 2 CUP and T_n_N_0-X_M_1_ in breast cancer patients.

|  |  | Type 2 **CUP** | |  | **T_n_N_0-X_M_1_** |  | **P value** |
| --- | --- | --- | --- | --- | --- | --- | --- |
| **Parameters** | | 733 | 100.00% | | 8867 | 100.00% |  |
| **Marital status** | **Married** | 347 | 47.30% | | 3550 | 40.00% | <0.001 |
|  | Others | 386 | 52.70% | | 5317 | 60.00% |  |
| **Age (years)** | ＜65 | 330 | 45.00% | | 5142 | 58.00% | <0.001 |
|  | ≥65 | 403 | 55.00% | | 3725 | 42.00% |  |
| **Race** | White | 618 | 84.30% | | 6495 | 73.20% | <0.001 |
|  | Others | 115 | 15.70% | | 2372 | 26.80% |  |
| **Gender** | Male | 12 | 1.60% | | 104 | 1.20% | 0.287 |
|  | Female | 721 | 98.40% | | 8763 | 98.80% |  |
| **Year of diagnosis** | 2004-2008 | 285 | 38.90% | | 3633 | 41.00% | 0.274 |
|  | 2009-2014 | 448 | 61.10% | | 5234 | 59.00% |  |
| **N stage** | N0 | 298 | 40.70% | | 1742 | 19.60% | <0.001 |
|  | Nn | 222 | 30.30% | | 5979 | 67.40% |  |
|  | NX | 213 | 29.10% | | 1146 | 12.90% |  |
| **Surgery** | No | 609 | 83.10% | | 8369 | 94.40% | <0.001 |
|  | Yes | 124 | 16.90% | | 498 | 5.60% |  |
| **Radiation** | No | 530 | 72.30% | | 6184 | 69.70% | 0.154 |
|  | Yes | 203 | 27.70% | | 2683 | 30.30% |  |
| **Chemotherapy** | No | 457 | 62.30% | | 4303 | 48.50% | <0.001 |
|  | Yes | 276 | 37.70% | | 4564 | 51.50% |  |

Table S6. Analysis of overall survival (OS) in type 2 CUP in breast cancer patients.

| **Parameter** |  |  |  | **Univariate analysis** | | **Multivariate analysis** | |
| --- | --- | --- | --- | --- | --- | --- | --- |
|  |  | Number | % | HR(95% CI) | P value | HR(95% CI) | P value |
| **Marital status** | **Married** | 347 | 47.30% | 0.834(0.696-0.998) | 0.048 | 0.85(0.704-1.025) | 0.09 |
|  | Others | 386 | 52.70% | 1(Referent) |  | 1(Referent) |  |
| **Age (years)** | ＜65 | 330 | 45.00% | 0.833(0.695-0.998) | 0.047 | 0.869(0.715-1.056) | 0.159 |
|  | ≥65 | 403 | 55.00% | 1(Referent) |  | 1(Referent) |  |
| **Race** | White | 618 | 84.30% | 0.871(0.678-1.121) | 0.283 | 0.82(0.631-1.067) | 0.139 |
|  | Others | 115 | 15.70% | 1(Referent) |  | 1(Referent) |  |
| **Gender** | Male | 12 | 1.60% | 0.995(0.445-2.228) | 0.991 | 1.131(0.496-2.58) | 0.77 |
|  | Female | 721 | 98.40% | 1(Referent) |  | 1(Referent) |  |
| **Year of diagnosis** | 2004-2008 | 285 | 38.90% | 1.138(0.942-1.375) | 0.18 | 1.392(1.077-1.799) | 0.011 |
|  | 2009-2014 | 448 | 61.10% | 1(Referent) |  | 1(Referent) |  |
| **N stage** | N0 | 298 | 40.70% | 1(Referent) |  | 1(Referent) |  |
|  | Nn | 222 | 30.30% | 0.985(0.765-1.200) | 0.709 | 0.944(0.747-1.192) | 0.628 |
|  | NX | 213 | 29.10% | 0.335(1.079-1.651) | 0.008 | 1.268(1.016-1.583) | 0.036 |
| **Surgery** | No | 609 | 83.10% | 1.481(1.152-1.905) | 0.002 | 1.408(1.087-1.825) | 0.01 |
|  | Yes | 124 | 16.90% | 1(Referent) |  | 1(Referent) |  |
| **Radiation** | No | 530 | 72.30% | 0.987(0.808-1.206) | 0.898 | 0.987(0.8-1.217) | 0.9 |
|  | Yes | 203 | 27.70% | 1(Referent) |  | 1(Referent) |  |
| **Chemotherapy** | No | 457 | 62.30% | 1.132(0.94-1.364) | 0.19 | 1.114(0.912-1.361) | 0.29 |
|  | Yes | 276 | 37.70% | 1(Referent) |  | 1(Referent) |  |

Table S7. Comparison of the clinical parameters of type 2 CUP and T_n_N_0-X_M_1_ in pancreatic cancer patients.

|  |  | **Type 2 CUP** |  | **T_n_N_0-X_M_1_** |  | **P value** |
| --- | --- | --- | --- | --- | --- | --- |
| **parameters** | | 563 | 100.00% | 3376 | 100.00% |  |
| **Marital status** | **Married** | 296 | 52.60% | 1712 | 50.70% | 0.439 |
|  | Others | 267 | 47.40% | 1664 | 49.30% |  |
| **Age (years)** | ＜65 | 190 | 33.70% | 1193 | 35.30% | 0.475 |
|  | ≥65 | 373 | 66.30% | 2183 | 64.70% |  |
| **Race** | White | 472 | 83.80% | 2650 | 78.50% | 0.003 |
|  | Others | 91 | 16.20% | 726 | 21.50% |  |
| **Gender** | Male | 293 | 52.00% | 1737 | 51.50% | 0.82 |
|  | Female | 270 | 48.00% | 1639 | 48.50% |  |
| **Year of diagnosis** | 2004-2008 | 227 | 40.30% | 1356 | 40.20% | 0.963 |
|  | 2009-2014 | 336 | 59.70% | 2020 | 59.80% |  |
| **N stage** | N0 | 292 | 51.90% | 1489 | 44.10% | <0.001 |
|  | Nn | 128 | 22.70% | 1095 | 32.40% |  |
|  | NX | 143 | 25.40% | 792 | 23.50% |  |
| **Surgery** | No | 511 | 90.80% | 3169 | 93.90% | 0.008 |
|  | Yes | 52 | 9.20% | 207 | 6.10% |  |
| **Radiation** | No | 526 | 93.40% | 3212 | 95.10% | 0.097 |
|  | Yes | 37 | 6.60% | 164 | 4.90% |  |
| **Chemotherapy** | No | 331 | 58.80% | 2007 | 59.40% | 0.781 |
|  | Yes | 232 | 41.20% | 1369 | 40.60% |  |

Table S8. Analysis of overall survival (OS) in type 2 CUP in pancreatic cancer patients.

| **Parameter** |  |  |  | **Univariate analysis** | | **Multivariate analysis** | |
| --- | --- | --- | --- | --- | --- | --- | --- |
|  |  | Number | % | HR(95% CI) | P value | HR(95% CI) | P value |
| **Marital status** | **Married** | 296 | 52.60% | 1.102(0.93-1.305) | 0.263 | 1.024(0.853-1.229) | 0.802 |
|  | Others | 267 | 47.40% | 1(Referent) |  | 1(Referent) |  |
| **Age (years)** | ＜65 | 190 | 33.70% | 0.716(0.597-0.859) | <0.001 | 0.817(0.674-0.989) | 0.038 |
|  | ≥65 | 373 | 66.30% | 1(Referent) |  | 1(Referent) |  |
| **Race** | White | 472 | 83.80% | 1.029(0.817-1.295) | 0.809 | 1.066(0.843-1.347) | 0.595 |
|  | Others | 91 | 16.20% | 1(Referent) |  | 1(Referent) |  |
| **Gender** | Male | 293 | 52.00% | 1.121(0.946-1.328) | 0.188 | 1.169(0.976-1.399) | 0.09 |
|  | Female | 270 | 48.00% | 1(Referent) |  | 1(Referent) |  |
| **Year of diagnosis** | 2004-2008 | 227 | 40.30% | 0.902(0.759-1.071) | 0.239 | 0.998(0.835-1.193) | 0.984 |
|  | 2009-2014 | 336 | 59.70% | 1(Referent) |  | 1(Referent) |  |
| **N stage** | N0 | 292 | 51.90% | 1(Referent) |  | 1(Referent) |  |
|  | Nn | 128 | 22.70% | 0.963(0.777-1.192) | 0.727 | 1.078(0.866-1.343) | 0.5 |
|  | NX | 143 | 25.40% | 1.026(0.837-1.258) | 0.803 | 0.981(0.793-1.213) | 0.858 |
| **Surgery** | No | 511 | 90.80% | 1.133(0.846-1.517) | 0.401 | 1.21(0.9-1.627) | 0.207 |
|  | Yes | 52 | 9.20% | 1(Referent) |  | 1(Referent) |  |
| **Radiation** | No | 526 | 93.40% | 1.213(0.864-1.702) | 0.265 | 1.141(0.811-1.606) | 0.448 |
|  | Yes | 37 | 6.60% | 1(Referent) |  | 1(Referent) |  |
| **Chemotherapy** | No | 331 | 58.80% | 2.465(2.054-2.959) | <0.001 | 2.438(2.013-2.954) | <0.001 |
|  | Yes | 232 | 41.20% | 1(Referent) |  | 1(Referent) |  |

Table S9. Comparison of the clinical parameters of type 2 CUP and T_n_N_0-X_M_1_ in colon cancer patients.

|  |  | **Type 2 CUP** |  | **T_n_N_0-X_M_1_** |  | **P value** |
| --- | --- | --- | --- | --- | --- | --- |
| **parameters** | | 469 | 100.00% | 1125 | 100.00% |  |
| **Marital status** | **Married** | 233 | 49.70% | 514 | 45.70% | 0.152 |
|  | Others | 236 | 50.30% | 611 | 54.30% |  |
| **Age (years)** | ＜65 | 162 | 34.50% | 453 | 40.30% | 0.037 |
|  | ≥65 | 307 | 65.50% | 672 | 59.70% |  |
| **Race** | White | 370 | 78.90% | 836 | 74.30% | 0.55 |
|  | Others | 99 | 21.10% | 289 | 25.70% |  |
| **Gender** | Male | 241 | 51.40% | 568 | 50.50% | 0.783 |
|  | Female | 228 | 48.60% | 557 | 49.50% |  |
| **Year of diagnosis** | 2004-2008 | 158 | 33.70% | 517 | 46.00% | <0.001 |
|  | 2009-2014 | 311 | 66.30% | 608 | 54.00% |  |
| **N stage** | N0 | 281 | 59.90% | 474 | 42.10% | <0.001 |
|  | Nn | 46 | 9.80% | 416 | 37.00% |  |
|  | NX | 142 | 30.30% | 235 | 20.90% |  |
| **Surgery** | No | 404 | 86.10% | 964 | 85.70% | 0.441 |
|  | Yes | 65 | 13.90% | 161 | 14.30% |  |
| **Radiation** | No | 430 | 91.70% | 1077 | 95.70% | 0.001 |
|  | Yes | 39 | 8.30% | 48 | 4.30% |  |
| **Chemotherapy** | No | 261 | 55.70% | 641 | 57.00% | 0.333 |
|  | Yes | 208 | 44.30% | 484 | 43.00% |  |

Table S10. Analysis of overall survival (OS) in type 2 CUP in colon cancer patients.

| **Parameter** |  |  |  | **Univariate analysis** | | **Multivariate analysis** | |
| --- | --- | --- | --- | --- | --- | --- | --- |
|  |  | Number | % | HR(95% CI) | P value | HR(95% CI) | P value |
| **Marital status** | **Married** | 233 | 49.70% | 0.722(0.593-0.879) | 0.001 | 0.915(0.74-1.13) | 0.408 |
|  | Others | 236 | 50.30% | **1(Referent)** |  | **1(Referent)** |  |
| **Age (years)** | ＜65 | 162 | 34.50% | 0.689(0.557-0.851) | 0.001 | 0.765(0.614-0.952) | 0.016 |
|  | ≥65 | 307 | 65.50% | **1(Referent)** |  | **1(Referent)** |  |
| **Race** | White | 370 | 78.90% | 1.19(0.93-1.522) | 0.166 | 1.162(0.903-1.496) | 0.244 |
|  | Others | 99 | 21.10% | **1(Referent)** |  | **1(Referent)** |  |
| **Gender** | Male | 241 | 51.40% | 0.953(0.783-1.159) | 0.628 | 0.973(0.79-1.199) | 0.8 |
|  | Female | 228 | 48.60% | **1(Referent)** |  | **1(Referent)** |  |
| **Year of diagnosis** | 2004-2008 | 158 | 33.70% | 1.506(1.231-1.843) | <0.001 | 1.333(1.083-1.642) | 0.007 |
|  | 2009-2014 | 311 | 66.30% | **1(Referent)** |  | **1(Referent)** |  |
| **N stage** | N0 | 281 | 59.90% | **1(Referent)** |  | **1(Referent)** |  |
|  | Nn | 46 | 9.80% | 0.973(0.690-1.371) | 0.875 | 1.162(0.820-1.646) | 0.4 |
|  | NX | 142 | 30.30% | 0.155(0.942-1.451) | 0.155 | 1.157(0.926-1.447) | 0.199 |
| **Surgery** | No | 404 | 86.10% | 2.351(1.694-3.264) | <0.001 | 2.985(2.118-4.206) | <0.001 |
|  | Yes | 65 | 13.90% | **1(Referent)** |  | **1(Referent)** |  |
| **Radiation** | No | 430 | 91.70% | 1.131(0.795-1.609) | 0.493 | 1.034(0.719-1.486) | 0.857 |
|  | Yes | 39 | 8.30% | **1(Referent)** |  | **1(Referent)** |  |
| **Chemotherapy** | No | 261 | 55.70% | 2.273(1.852-2.789) | <0.001 | 2.495(2.003-3.109) | <0.001 |
|  | Yes | 208 | 44.30% | **1(Referent)** |  | **1(Referent)** |  |

Table S11. Comparison of the clinical parameters of type 2 CUP and T_n_N_0-X_M_1_ in ovary cancer patients.

|  |  | Type 2 **CUP** |  | **T_n_N_0-X_M_1_** |  | **P value** |
| --- | --- | --- | --- | --- | --- | --- |
| **parameters** | | 288 | 100.00% | 12789 | 100.00% |  |
| **Marital status** | **Married** | 98 | 34.00% | 5886 | 46.00% | <0.001 |
|  | Others | 190 | 66.00% | 6903 | 54.00% |  |
| **Age** | ＜65 | 72 | 25.00% | 6032 | 47.20% | <0.001 |
|  | ≥65 | 216 | 75.00% | 6757 | 52.80% |  |
| **Race** | White | 237 | 82.30% | 10563 | 82.60% | 0.882 |
|  | Others | 51 | 17.70% | 2226 | 17.40% |  |
| **Year of diagnosis** | 2004-2008 | 100 | 34.70% | 5895 | 46.10% | <0.001 |
|  | 2009-2014 | 188 | 65.30% | 6894 | 53.90% |  |
| **N stage** | N0 | 175 | 60.80% | 6257 | 48.90% | <0.001 |
|  | Nn | 35 | 12.20% | 3962 | 31.00% |  |
|  | NX | 78 | 27.10% | 2570 | 20.10% |  |
| **Surgery** | No | 255 | 88.50% | 10376 | 81.10% | 0.001 |
|  | Yes | 33 | 11.50% | 2413 | 18.90% |  |
| **Radiation** | No | 279 | 96.90% | 12591 | 98.50% | 0.049 |
|  | Yes | 9 | 3.10% | 198 | 1.50% |  |
| **Chemotherapy** | No | 121 | 42.00% | 3614 | 28.30% | <0.001 |
|  | Yes | 167 | 58.00% | 9175 | 71.70% |  |

Table S12, Analysis of overall survival (OS) in type 2 CUP in ovary cancer patients.

| **Parameter** |  |  |  | **Univariate analysis** | | **Multivariate analysis** | |
| --- | --- | --- | --- | --- | --- | --- | --- |
|  |  | Number | % | HR(95% CI) | P value | HR(95% CI) | P value |
| **Marital status** | **Married** | 98 | 34.00% | 0.908(0.691-1.194) | 0.49 | 1.17(0.88-1.556) | 0.281 |
|  | Others | 190 | 66.00% | **1(Referent)** |  | **1(Referent)** |  |
| **Age** | ＜65 | 72 | 25.00% | 0.646(0.473-0.883) | 0.006 | 0.665(0.482-0.917) | 0.013 |
|  | ≥65 | 216 | 75.00% | **1(Referent)** |  | **1(Referent)** |  |
| **Race** | White | 237 | 82.30% | 1.049(0.748-1.472) | 0.779 | 0.996(0.706-1.405) | 0.98 |
|  | Others | 51 | 17.70% | **1(Referent)** |  | **1(Referent)** |  |
| **Year of diagnosis** | 2004-2008 | 100 | 34.70% | 1.268(0.973-1.652) | 0.079 | 1.157(0.882-1.518) | 0.292 |
|  | 2009-2014 | 188 | 65.30% | **1(Referent)** |  | **1(Referent)** |  |
| **N stage** | N0 | 175 | 60.80% | **1(Referent)** |  | **1(Referent)** |  |
|  | Nn | 35 | 12.20% | 0.859(0.555-1.382) | 0.494 | 0.984(0.629-1.542) | 0.946 |
|  | NX | 78 | 27.10% | 1.540(1.148-2.066) | 0.014 | 1.467(1.082-1.988) | 0.102 |
| **Surgery** | No | 255 | 88.50% | 1.491(0.989-2.248) | 0.056 | 1.436(0.944-2.184) | 0.091 |
|  | Yes | 33 | 11.50% | **1(Referent)** |  | **1(Referent)** |  |
| **Radiation** | No | 279 | 96.90% | 0.894(0.441-1.815) | 0.757 | 1.094(0.523-2.292) | 0.811 |
|  | Yes | 9 | 3.10% | **1(Referent)** |  | **1(Referent)** |  |
| **Chemotherapy** | No | 121 | 42.00% | 3.075(2.341-4.039) | <0.001 | 3.101(2.333-4.123) | <0.001 |
|  | Yes | 167 | 58.00% | **1(Referent)** |  | **1(Referent)** |  |

Table S13. Comparison of the clinical parameters of type 2 CUP and T_n_N_0-X_M_1_ in **prostate gland** cancer patients.

|  |  | **Type 2 CUP** |  | **T_n_N_0-X_M_1_** |  | **P value** |
| --- | --- | --- | --- | --- | --- | --- |
| **parameters** | | 228 | 100.00% | 19910 | 100.00% |  |
| **Marital status** | **Married** | 129 | 56.60% | 11410 | 57.30% | 0.841 |
|  | Others | 99 | 43.40% | 8500 | 42.70% |  |
| **Age** | ＜65 | 48 | 21.10% | 6136 | 30.80% | 0.002 |
|  | ≥65 | 180 | 78.90% | 13774 | 69.20% |  |
| **Race** | White | 177 | 77.60% | 14886 | 74.80% | 0.357 |
|  | Others | 51 | 22.40% | 5024 | 25.20% |  |
| **Year of diagnosis** | 2004-2008 | 58 | 25.40% | 8227 | 41.30% | <0.001 |
|  | 2009-2014 | 170 | 74.60% | 11683 | 58.70% |  |
| **N stage** | N0 | 130 | 57.00% | 11415 | 57.30% | 0.005 |
|  | Nn | 39 | 17.10% | 4768 | 23.90% |  |
|  | NX | 59 | 25.90% | 3727 | 18.70% |  |
| **Surgery** | No | 204 | 89.50% | 19189 | 96.40% | <0.001 |
|  | Yes | 24 | 10.50% | 721 | 3.60% |  |
| **Radiation** | No | 166 | 72.80% | 15448 | 77.60% | 0.091 |
|  | Yes | 62 | 27.20% | 4462 | 22.40% |  |
| **Chemotherapy** | No | 204 | 89.50% | 18464 | 92.70% | 0.066 |
|  | Yes | 24 | 10.50% | 1446 | 7.30% |  |

Table S14. Analysis of overall survival (OS) in type 2 CUP in **prostate gland** cancer patients.

| **Parameter** |  |  |  | **Univariate analysis** | | **Multivariate analysis** | |
| --- | --- | --- | --- | --- | --- | --- | --- |
|  |  | Number | % | HR(95% CI) | P value | HR(95% CI) | P value |
| **Marital status** | **Married** | 129 | 56.60% | 0.832(0.601-1.152) | 0.268 | 0.822(0.592-1.143) | 0.245 |
|  | Others | 99 | 43.40% | **1(Referent)** | | **1(Referent)** |  |
| **Age** | ＜65 | 48 | 21.10% | 0.539(0.345-0.841) | 0.006 | 0.553(0.353-0.867) | 0.01 |
|  | ≥65 | 180 | 78.90% | **1(Referent)** | | **1(Referent)** |  |
| **Race** | White | 177 | 77.60% | 1.165(0.783-1.733) | 0.453 | 1.277(0.853-1.912) | 0.235 |
|  | Others | 51 | 22.40% | **1(Referent)** | | **1(Referent)** |  |
| **Year of diagnosis** | 2004-2008 | 58 | 25.40% | 0.958(0.674-1.361) | 0.81 | 0.912(0.628-1.324) | 0.628 |
|  | 2009-2014 | 170 | 74.60% | **1(Referent)** | | **1(Referent)** |  |
| **N stage** | N0 | 130 | 57.00% | **1(Referent)** | | **1(Referent)** |  |
|  | Nn | 39 | 17.10% | 1.079(0.747-1.558) | 0.685 | 0.995(0.678-1.46) | 0.98 |
|  | NX | 59 | 25.90% | 0.846(0.504-1.421) | 0.527 | 0.797(0.465-1.367) | 0.41 |
| **Surgery** | No | 204 | 89.50% | 2.377(1.282-4.405) | 0.006 | 2.262(1.21-4.23) | 0.011 |
|  | Yes | 24 | 10.50% | **1(Referent)** | | **1(Referent)** |  |
| **Radiation** | No | 166 | 72.80% | 1.154(0.807-1.65) | 0.432 | 1.116(0.773-1.612) | 0.557 |
|  | Yes | 62 | 27.20% | **1(Referent)** | | **1(Referent)** |  |
| **Chemotherapy** | No | 204 | 89.50% | 1.35(0.747-2.437) | 0.32 | 1.331(0.729-2.431) | 0.351 |
|  | Yes | 24 | 10.50% | **1(Referent)** | | **1(Referent)** |  |

Table S15. Comparison of the clinical parameters of type 2 CUP and T_n_N_0-X_M_1_ in **kidney** cancer patients.

|  |  | **Type 2 CUP** |  | **T_n_N_0-X_M_1_** |  | **P value** |
| --- | --- | --- | --- | --- | --- | --- |
| **parameters** | | 144 | 100.00% | 14680 | 100.00% |  |
| **Marital status** | **Married** | 86 | 59.70% | 8570 | 58.40% | 0.797 |
|  | Others | 58 | 40.30% | 6110 | 41.60% |  |
| **Age** | ＜65 | 64 | 44.40% | 7407 | 50.50% | 0.155 |
|  | ≥65 | 80 | 55.60% | 7273 | 49.50% |  |
| **Race** | White | 114 | 79.20% | 12177 | 82.90% | 0.222 |
|  | Others | 30 | 20.80% | 2503 | 17.10% |  |
| **Sex** | Male | 103 | 71.50% | 9974 | 67.90% | 0.418 |
|  | Female | 41 | 28.50% | 4706 | 32.10% |  |
| **Year of diagnosis** | 2004-2008 | 42 | 29.20% | 6038 | 41.10% | 0.004 |
|  | 2009-2014 | 102 | 70.80% | 8642 | 58.90% |  |
| **N stage** | N0 | 88 | 61.10% | 8553 | 58.30% | <0.001 |
|  | Nn | 28 | 19.40% | 4599 | 31.30% |  |
|  | NX | 28 | 19.40% | 1528 | 10.40% |  |
| **Surgery** | No | 111 | 77.10% | 12777 | 87.00% | 0.001 |
|  | Yes | 33 | 22.90% | 1903 | 13.00% |  |
| **Radiation** | No | 96 | 66.70% | 10850 | 73.90% | 0.054 |
|  | Yes | 48 | 33.30% | 3830 | 26.10% |  |
| **Chemotherapy** | No | 84 | 58.30% | 8422 | 57.40% | 0.864 |
|  | Yes | 60 | 41.70% | 6258 | 42.60% |  |

Table S16. Analysis of overall survival (OS) in type 2 CUP in **kidney** cancer patients.

| **Parameter** |  |  |  | **Univariate analysis** | | **Multivariate analysis** | |
| --- | --- | --- | --- | --- | --- | --- | --- |
|  |  | Number | % | HR(95% CI) | P value | HR(95% CI) | P value |
| **Marital status** | **Married** | 86 | 59.70% | 1.062(0.73-1.547) | 0.752 | 1.198(0.764-1.877) | 0.431 |
|  | Others | 58 | 40.30% | **1(Referent)** |  | **1(Referent)** |  |
| **Age (years)** | ＜65 | 64 | 44.40% | 0.496(0.341-0.723) | <0.001 | 0.471(0.31-0.716) | <0.001 |
|  | ≥65 | 80 | 55.60% | **1(Referent)** |  | **1(Referent)** |  |
| **Race** | White | 114 | 79.20% | 1.164(0.725-1.868) | 0.529 | 0.881(0.511-1.52) | 0.649 |
|  | Others | 30 | 20.80% | **1(Referent)** |  | **1(Referent)** |  |
| **Gender** | Male | 103 | 71.50% | 1.104(0.735-1.659) | 0.633 | 1.116(0.726-1.715) | 0.617 |
|  | Female | 41 | 28.50% | **1(Referent)** |  | **1(Referent)** |  |
| **Year of diagnosis** | 2004-2008 | 42 | 29.20% | 1.277(0.869-1.878) | 0.213 | 1.321(0.841-2.073) | 0.227 |
|  | 2009-2014 | 102 | 70.80% | **1(Referent)** |  | **1(Referent)** |  |
| **N stage** | N0 | 88 | 61.10% | **1(Referent)** |  | **1(Referent)** |  |
|  | Nn | 28 | 19.40% | 1.624(1.018-2.590) | 0.042 | 1.8(1.11-2.92) | 0.17 |
|  | NX | 28 | 19.40% | 1.238(0.790-1.939) | 0.352 | 0.872(0.541-1.403) | 0.571 |
| **Surgery** | No | 111 | 77.10% | 1.336(0.867-2.057) | 0.189 | 1.182(0.742-1.884) | 0.481 |
|  | Yes | 33 | 22.90% | **1(Referent)** |  | **1(Referent)** |  |
| **Radiation** | No | 96 | 66.70% | 1.403(0.946-2.08) | 0.092 | 1.363(0.904-2.055) | 0.14 |
|  | Yes | 48 | 33.30% | **1(Referent)** |  | **1(Referent)** |  |
| **Chemotherapy** | No | 84 | 58.30% | 1.637(1.123-2.385) | 0.01 | 1.535(0.991-2.378) | 0.055 |
|  | Yes | 60 | 41.70% | **1(Referent)** |  | **1(Referent)** |  |

Table S17. Comparison of the clinical parameters of type 2 CUP and T_n_N_0-X_M_1_ in **testis** cancer patients.

|  |  | **Type 2 CUP** |  | **T_n_N_0-X_M_1_** |  | **P value** |
| --- | --- | --- | --- | --- | --- | --- |
|  |  | 117 | 100.00% | 1133 | 100.00% |  |
| **Marital status** | **Married** | 46 | 39.30% | 329 | 29.00% | 0.026 |
|  | Others | 71 | 60.70% | 804 | 71.00% |  |
| **Age** | ＜65 | 111 | 94.90% | 1114 | 98.30% | 0.024 |
|  | ≥65 | 6 | 5.10% | 19 | 1.70% |  |
| **Race** | White | 110 | 94.00% | 1022 | 90.20% | 0.243 |
|  | Others | 7 | 6.00% | 111 | 9.80% |  |
| **Year of diagnosis** | 2004-2008 | 56 | 47.90% | 495 | 43.70% | 0.434 |
|  | 2009-2014 | 61 | 52.10% | 638 | 56.30% |  |
| **N stage** | N0 | 45 | 38.50% | 407 | 35.90% | 0.008 |
|  | Nn | 56 | 47.90% | 653 | 57.60% |  |
|  | NX | 16 | 13.70% | 73 | 6.40% |  |
| **Surgery** | No | 83 | 70.90% | 1017 | 89.80% | <0.001 |
|  | Yes | 34 | 29.10% | 116 | 10.20% |  |
| **Radiation** | No | 104 | 88.90% | 1063 | 93.80% | 0.05 |
|  | Yes | 13 | 11.10% | 70 | 6.20% |  |
| **Chemotherapy** | No | 10 | 8.50% | 95 | 8.40% | 0.863 |
|  | Yes | 107 | 91.50% | 1038 | 91.60% |  |

Table S18. Analysis of overall survival (OS) in type 2 CUP in **testis** cancer patients.

| **Parameter** |  |  |  | **Univariate analysis** | | **Multivariate analysis** | |
| --- | --- | --- | --- | --- | --- | --- | --- |
|  |  | Number | % | HR(95% CI) | P value | HR(95% CI) | P value |
| **Marital status** | **Married** | 46 | 39.30% | 1.3(0.708-2.388) | 0.397 | 1.33(0.675-2.62) | 0.409 |
|  | Others | 71 | 60.70% | **1(Referent)** |  | **1(Referent)** |  |
| **Age** | ＜65 | 111 | 94.90% | 0.41(0.146-1.149) | 0.09 | 0.74(0.178-3.071) | 0.678 |
|  | ≥65 | 6 | 5.10% | **1(Referent)** |  | **1(Referent)** |  |
| **Race** | White | 110 | 94.00% | 0.403(0.158-1.028) | 0.057 | 0.378(0.127-1.127) | 0.081 |
|  | Others | 7 | 6.00% | **1(Referent)** |  | **1(Referent)** |  |
| **Year of diagnosis** | 2004-2008 | 56 | 47.90% | 1.337(0.725-2.464) | 0.352 | 1.398(0.688-2.837) | 0.354 |
|  | 2009-2014 | 61 | 52.10% | **1(Referent)** |  | **1(Referent)** |  |
| **N stage** | N0 | 45 | 38.50% | **1(Referent)** |  | **1(Referent)** | 0.835 |
|  | Nn | 56 | 47.90% | 0.775(0.316-1.902) | 0.578 | 1.119(0.411-3.047) | 0.826 |
|  | NX | 16 | 13.70% | 0.784(0.331-1.855) | 0.58 | 0.989(0.371-2.635) | 0.983 |
| **Surgery** | No | 83 | 70.90% | 2.481(1.102-5.588) | 0.028 | 2.856(1.211-6.736) | 0.016 |
|  | Yes | 34 | 29.10% | **1(Referent)** |  | **1(Referent)** |  |
| **Radiation** | No | 104 | 88.90% | 0.409(0.195-0.856) | 0.018 | 0.457(0.195-1.072) | 0.072 |
|  | Yes | 13 | 11.10% | **1(Referent)** |  | **1(Referent)** |  |
| **Chemotherapy** | No | 10 | 8.50% | 2.532(1.065-6.021) | 0.036 | 2.228(0.789-6.288) | 0.13 |
|  | Yes | 107 | 91.50% | **1(Referent)** |  | **1(Referent)** |  |

Table S19. Comparison of the clinical parameters of type 2 CUP and T_n_N_0-X_M_1_ in **biliary tract** cancer patients.

|  |  | **Type 2 CUP** |  | **T_n_N_0-X_M_1_** |  | **P value** |
| --- | --- | --- | --- | --- | --- | --- |
|  |  | 104 | 100.00% | 292 | 100.00% |  |
| **Marital status** | **Married** | 59 | 56.70% | 151 | 51.70% | 0.424 |
|  | Others | 45 | 43.30% | 141 | 48.30% |  |
| **Age** | ＜65 | 47 | 45.20% | 136 | 46.60% | 0.82 |
|  | ≥65 | 57 | 54.80% | 156 | 53.40% |  |
| **Race** | White | 85 | 81.70% | 240 | 82.20% | 0.883 |
|  | Others | 19 | 18.30% | 52 | 17.80% |  |
| **Gender** | Male | 49 | 47.10% | 145 | 49.70% | 0.732 |
|  | Female | 55 | 52.90% | 147 | 50.30% |  |
| **Year of diagnosis** | 2004-2008 | 30 | 28.80% | 103 | 35.30% | 0.277 |
|  | 2009-2014 | 74 | 71.20% | 189 | 64.70% |  |
| **N stage** | N0 | 59 | 56.70% | 136 | 46.60% | 0.001 |
|  | Nn | 17 | 16.30% | 105 | 36.00% |  |
|  | NX | 28 | 26.90% | 51 | 17.50% |  |
| **Surgery** | No | 95 | 91.30% | 277 | 94.90% | 0.231 |
|  | Yes | 9 | 8.70% | 15 | 5.10% |  |
| **Radiation** | No | 98 | 94.20% | 261 | 89.40% | 1 |
|  | Yes | 6 | 5.80% | 31 | 10.60% |  |
| **Chemotherapy** | No | 50 | 48.10% | 142 | 48.60% | 1 |
|  | Yes | 54 | 51.90% | 150 | 51.40% |  |

Table S20. Analysis of overall survival (OS) in type 2 CUP in **biliary tract** cancer patients

| **Parameter** |  |  |  | **Univariate analysis** | | **Multivariate analysis** | |
| --- | --- | --- | --- | --- | --- | --- | --- |
|  |  | Number | % | HR(95% CI) | P value | HR(95% CI) | P value |
| **Marital status** | **Married** | 59 | 56.70% | 0.799(0.534-1.196) | 0.276 | 1.059(0.665-1.688) | 0.809 |
|  | Others | 45 | 43.30% | **1(Referent)** | | **1(Referent)** |  |
| **Age** | ＜65 | 47 | 45.20% | 0.684(0.455-1.03) | 0.069 | 0.859(0.533-1.387) | 0.535 |
|  | ≥65 | 57 | 54.80% | **1(Referent)** | | **1(Referent)** |  |
| **Race** | White | 85 | 81.70% | 0.914(0.546-1.532) | 0.734 | 0.799(0.459-1.392) | 0.428 |
|  | Others | 19 | 18.30% | **1(Referent)** | | **1(Referent)** |  |
| **Gender** | Male | 49 | 47.10% | 1.122(0.745-1.689) | 0.583 | 1.36(0.851-2.173) | 0.198 |
|  | Female | 55 | 52.90% | **1(Referent)** | | **1(Referent)** |  |
| **Year of diagnosis** | 2004-2008 | 30 | 28.80% | 1.105(0.712-1.714) | 0.656 | 0.912(0.572-1.454) | 0.7 |
|  | 2009-2014 | 74 | 71.20% | **1(Referent)** |  | **1(Referent)** |  |
| **N stage** | N0 | 59 | 56.70% | **1(Referent)** | | **1(Referent)** |  |
|  | Nn | 17 | 16.30% | 0.846(0.533-1.342) | 0.477 | 0.708(0.43-1.164) | 0.173 |
|  | NX | 28 | 26.90% | 0.813(0.427-1.546) | 0.527 | 0.925(0.462-1.852) | 0.826 |
| **Surgery** | No | 95 | 91.30% | 0.862(0.432-1.719) | 0.673 | 0.583(0.272-1.253) | 0.167 |
|  | Yes | 9 | 8.70% | **1(Referent)** | | **1(Referent)** |  |
| **Radiation** | No | 98 | 94.20% | 0.986(0.43-2.258) | 0.973 | 1.15(0.48-2.757) | 0.754 |
|  | Yes | 6 | 5.80% | **1(Referent)** | | **1(Referent)** |  |
| **Chemotherapy** | No | 50 | 48.10% | 2.704(1.77-4.129) | 0 | 3.123(1.882-5.182) | 0 |
|  | Yes | 54 | 51.90% | **1(Referent)** |  | **1(Referent)** |  |

Table S21. Comparison of the clinical parameters of type 2 CUP and T_n_N_0-X_M_1_ in **stomach** cancer patients.

|  |  | **Type 2 CUP** |  | **T_n_N_0-X_M_1_** |  | **P value** |
| --- | --- | --- | --- | --- | --- | --- |
|  |  | 71 | 100.00% | 2151 | 100.00% |  |
| **Marital status** | **Married** | 39 | 54.90% | 1173 | 54.50% | 1 |
|  | Others | 32 | 45.10% | 978 | 45.50% |  |
| **Age** | ＜65 | 32 | 45.10% | 1048 | 48.70% | 0.63 |
|  | ≥65 | 39 | 54.90% | 1103 | 51.30% |  |
| **Race** | White | 47 | 66.20% | 1504 | 69.90% | 0.512 |
|  | Others | 24 | 33.80% | 647 | 30.10% |  |
| **Gender** | Male | 34 | 47.90% | 1187 | 55.20% | 0.228 |
|  | Female | 37 | 52.10% | 964 | 44.80% |  |
| **Year of diagnosis** | 2004-2008 | 20 | 28.20% | 1020 | 47.40% | 0.002 |
|  | 2009-2014 | 51 | 71.80% | 1131 | 52.60% |  |
| **N stage** | N0 | 49 | 69.00% | 850 | 39.50% | <0.001 |
|  | Nn | 7 | 9.90% | 866 | 40.30% |  |
|  | NX | 15 | 21.10% | 435 | 20.20% |  |
| **Surgery** | No | 53 | 74.60% | 1902 | 88.40% | 0.001 |
|  | Yes | 18 | 25.40% | 249 | 11.60% |  |
| **Radiation** | No | 63 | 88.70% | 1950 | 90.70% | 0.536 |
|  | Yes | 8 | 11.30% | 201 | 9.30% |  |
| **Chemotherapy** | No | 44 | 62.00% | 1170 | 54.40% | 0.227 |
|  | Yes | 27 | 38.00% | 981 | 45.60% |  |

Table S22. Analysis of overall survival (OS) in type 2 CUP in **stomach** cancer patients.

| **Parameter** |  |  |  | **Univariate analysis** | | **Multivariate analysis** | |
| --- | --- | --- | --- | --- | --- | --- | --- |
|  |  | Number | % | HR(95% CI) | P value | HR(95% CI) | P value |
| **Marital status** | **Married** | 39 | 54.90% | 0.826(0.503-1.354) | 0.448 | 0.877(0.52-1.481) | 0.625 |
|  | Others | 32 | 45.10% | **1(Referent)** | | **1(Referent)** |  |
| **Age** | ＜65 | 32 | 45.10% | 0.727(0.441-1.2) | 0.213 | 0.745(0.412-1.349) | 0.331 |
|  | ≥65 | 39 | 54.90% | **1(Referent)** | | **1(Referent)** |  |
| **Race** | White | 47 | 66.20% | 0.864(0.508-1.47) | 0.59 | 0.902(0.5-1.627) | 0.731 |
|  | Others | 24 | 33.80% | **1(Referent)** | | **1(Referent)** |  |
| **Gender** | Male | 34 | 47.90% | 1.034(0.629-1.703) | 0.894 | 1.112(0.63-1.961) | 0.715 |
|  | Female | 37 | 52.10% | **1(Referent)** | | **1(Referent)** |  |
| **Year of diagnosis** | 2004-2008 | 20 | 28.20% | 1.251(0.737-2.125) | 0.406 | 1.272(0.719-2.252) | 0.409 |
|  | 2009-2014 | 51 | 71.80% | **1(Referent)** |  | **1(Referent)** |  |
| **N stage** | N0 | 49 | 69.00% | **1(Referent)** | | **1(Referent)** |  |
|  | Nn | 7 | 9.90% | 1.471(0.655-3.305) | 0.350 | 1.4(0.596-3.290) | 0.440 |
|  | NX | 15 | 21.10% | 2.007(1.042-3.868) | 0.037 | 1.478(0.712-3.070) | 0.294 |
| **Surgery** | No | 53 | 74.60% | 1.267(0.717-2.242) | 0.415 | 1.278(0.676-2.415) | 0.45 |
|  | Yes | 18 | 25.40% | **1(Referent)** | | **1(Referent)** |  |
| **Radiation** | No | 63 | 88.70% | 1.122(0.507-2.483) | 0.776 | 0.942(0.383-2.317) | 0.896 |
|  | Yes | 8 | 11.30% | **1(Referent)** | | **1(Referent)** |  |
| **Chemotherapy** | No | 44 | 62.00% | 2.173(1.283-3.682) | 0.004 | 1.983(1.112-3.539) | 0.02 |
|  | Yes | 27 | 38.00% | **1(Referent)** |  | **1(Referent)** |  |
